# Supplementary material for: Mechanical Function of the Nucleus in Force Generation during Epithelial Morphogenesis
Source: Dev Cell. 2019 Jul 22;50(2):197–211.e5. doi: 10.1016/j.devcel.2019.05.027 (PMC6658619; doi:10.1016/j.devcel.2019.05.027)
Supplement: Document S1. Figures S1–S4 [file mmc1.pdf]

Developmental Cell, Volume *50*

Supplemental Information

Mechanical Function of the Nucleus in Force Generation during Epithelial Morphogenesis

Arnaud Ambrosini, Mégane Rayer, Bruno Monier, and Magali Suzanne

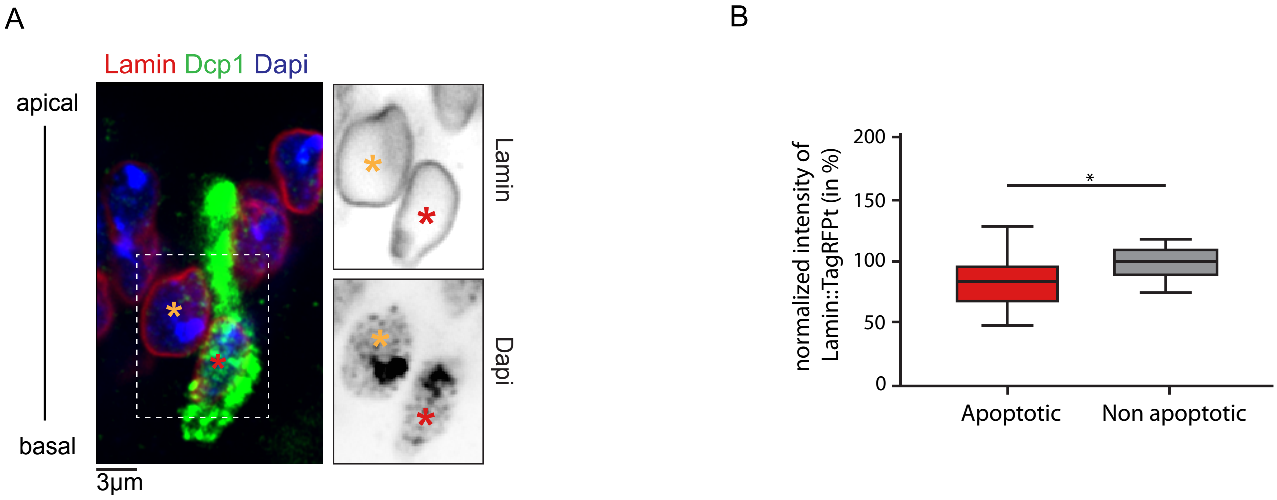

Figure S1. Nucleus morphology and integrity in early apoptotic cells
(A) Sagittal section of a fixed prepupal leg (left). Chromatin (DAPI) is in blue, nuclear envelope (Lamin) in red. An apoptotic cell is visualized by anti-Dcp1 staining (green). Close up views of Lamin (top) and DAPI (bottom) are shown on the right. Lamin distribution appears similar in apoptotic (red star) and non-apoptotic (orange star) nuclei. The apoptotic cell is at the initiation stage, before force generation. (B) Box plot of Lamin::RFPt levels in apoptotic (n=11) and non-apoptotic (n=35) nuclei. Apoptotic nuclei were selected on their basal localization, during or just prior the force generation stage. Mann Whitney test p value < 0.0159 (*). (Related to Figure 2).

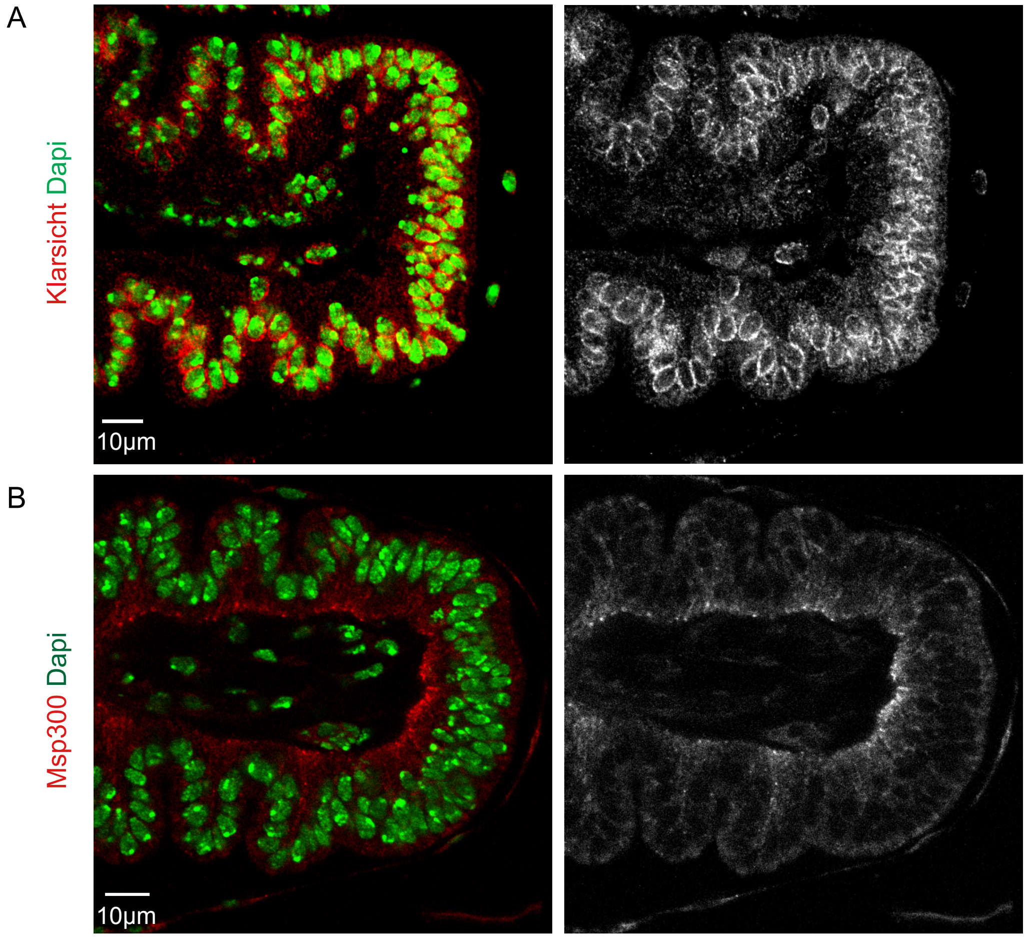

Figure S2. Localisation of KASH-domain proteins in the Drosophila leg
(A-B) Localization of the LINC members Klarsicht (A) and Msp-300 (B) in the leg tissue. Proteins of interest are shown in red and white and the nuclei in green. Msp-300 localisation is achieved using a Venus protein trap fly line. The Msp-300-Venus fusion protein is not perinuclear in the prepupal leg. By comparison, Klarsicht, which is detected by an antibody, is perinuclear in this tissue. Note that the black and white panel for Klarsicht in (A) is shown in purple in Fig. 3B. (Related to Fig. 3).

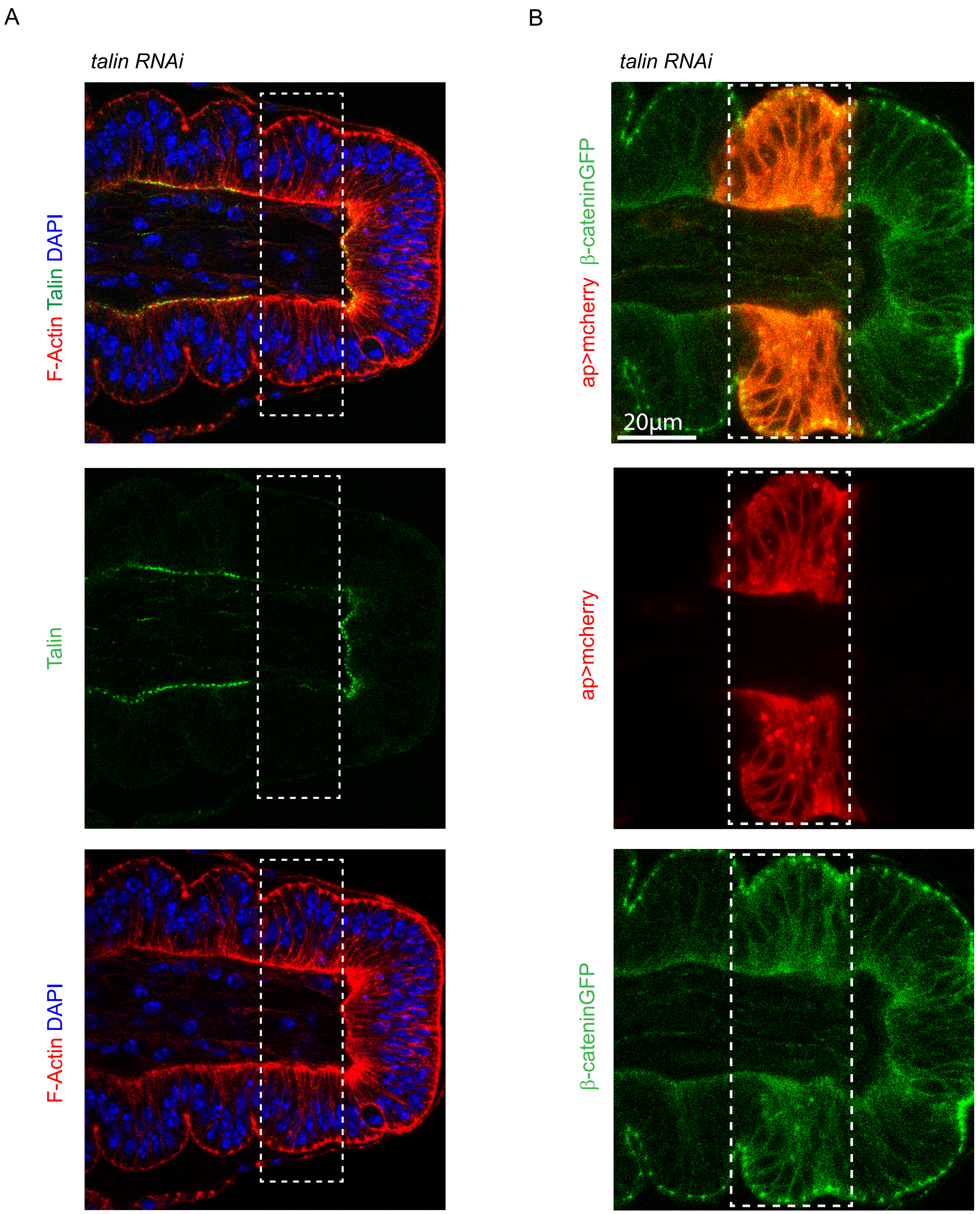

Figure S3. Talin inactivation does not alter tissue integrity in the prepupal leg
(A) Sagittal view of an apterous::Gal4; UAS::talin RNAi white pupa leg disc showing the distribution of Talin (green), F-actin organization (red) and nuclear positioning (blue). Note the strong reduction of Talin in the apterous domain (white dotted square). F-actin and nuclei positioning are unaltered in this domain. (B) Sagittal view of an apterous::Gal4; UAS::talin RNAi white pupal leg disc showing the distribution of β-catenin (green). Note that the epithelium is normally polarized in the apterous domain (white dotted square) in which *talin* is inactivated. UAS::mCherry labels the apterous domain. (Related to Figure 4)

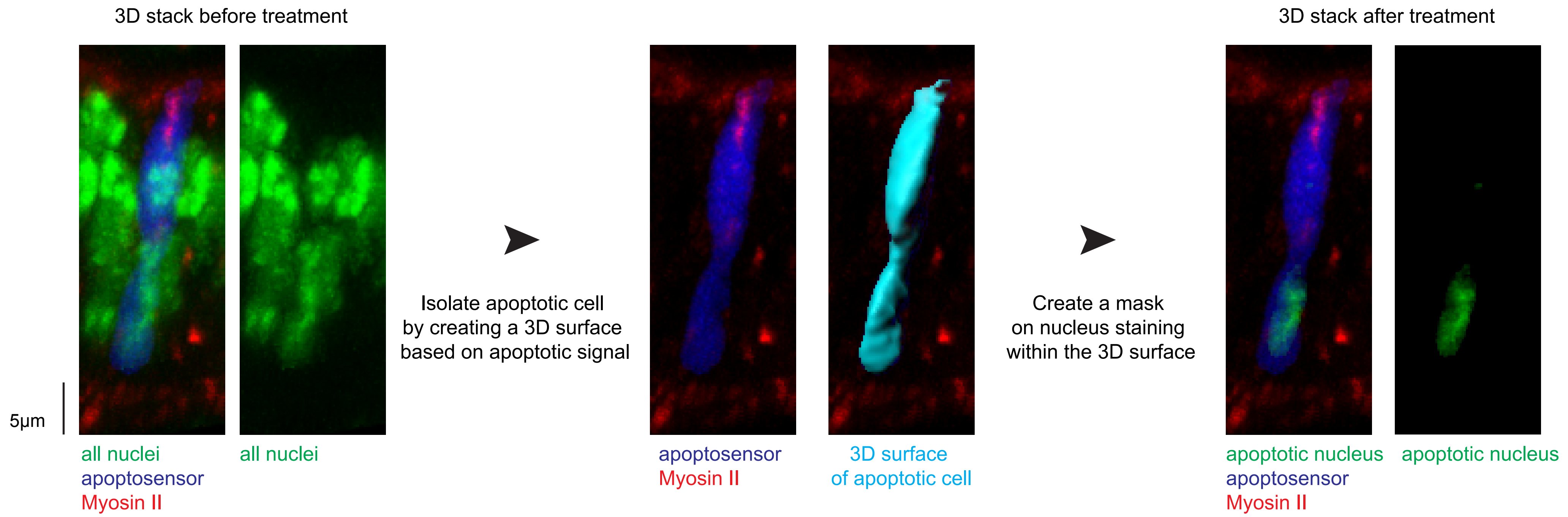

Figure S4. Post-acquisition method to isolate the apoptotic nucleus
Images recapitulating the different steps of apoptotic nucleus isolation from 3D reconstruction stack. 1. Isolation of an apoptotic cell by creating a 3D surface based on the expression of the apoptosensor (GC3Ai, blue). 2. Creation of a mask to extract nucleus staining (green) specifically in the apoptotic cell. Despite an originally crowded environment, this method allows to specifically visualize and track the apoptotic nucleus in live samples. (Related to Figure 5)
