# Supplementary material for: Mechanical Function of the Nucleus in Force Generation during Epithelial Morphogenesis
Source: Dev Cell. 2019 Jul 22;50(2):197–211.e5. doi: 10.1016/j.devcel.2019.05.027 (PMC6658619; doi:10.1016/j.devcel.2019.05.027)
Supplement: Document S2. Article plus Supplemental Information [file mmc8.pdf]

Developmental Cell

Article

Mechanical Function of the Nucleus in Force Generation during Epithelial Morphogenesis

Graphical Abstract

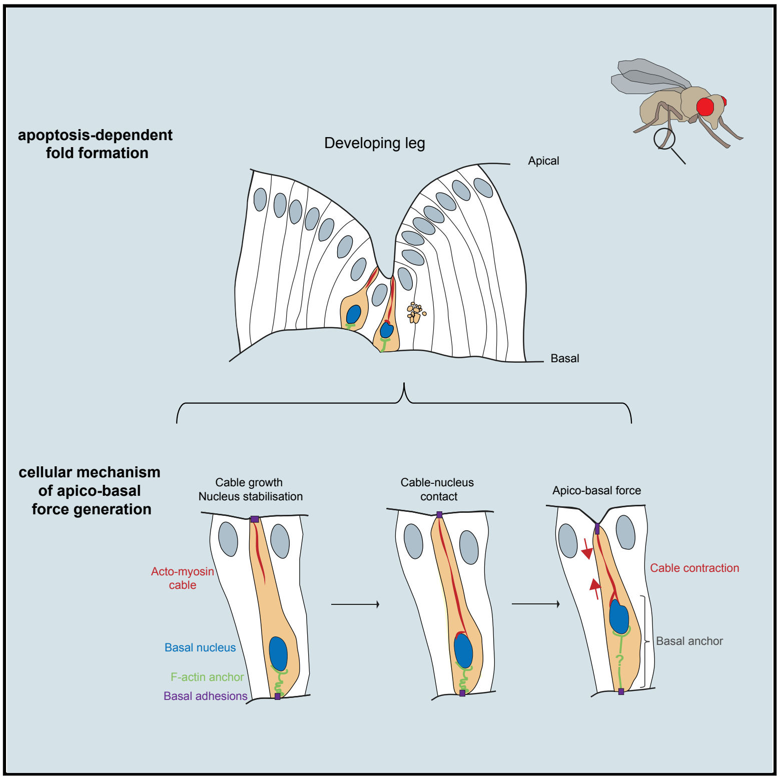

Highlights

- A contractile actomyosin structure connects the apical surface to the nucleus
- Nucleus anchoring to basal adhesion offers resistance to force generation
- The nucleus acts as a key component of the force-generation machinery
- Dying cell dynamics reveals a mechanism of apico-basal force generation

Authors

Arnaud Ambrosini, Mégane Rayer, Bruno Monier, Magali Suzanne

Correspondence

bruno.monier@univ-tlse3.fr (B.M.), magali.suzanne@univ-tlse3.fr (M.S.)

In Brief

Although essential for morphogenesis, apico-basal forces remain poorly understood. Ambrosini, Rayer, et al. reveal that, in dying cells, apico-basal forces rely on a dynamic actomyosin structure connecting the apical surface to the nucleus, itself anchored to basal adhesions. This work reveals the active contribution of the nucleus to force production.

Ambrosini et al., 2019, Developmental Cell 50, 197–211
July 22, 2019 © 2019 The Author(s). Published by Elsevier Inc.
https://doi.org/10.1016/j.devcel.2019.05.027

Developmental Cell
Article

Mechanical Function of the Nucleus in Force Generation during Epithelial Morphogenesis

Arnaud Ambrosini,[1,2] Mégane Rayer,[1,2] Bruno Monier,[1,*] and Magali Suzanne[1,3,*]
[1]LBCMCP, Centre de Biologie Intégrative (CBI), Université de Toulouse, CNRS, UPS, 118 route de Narbonne, 31062 Toulouse, France
[2]These authors contributed equally
[3]Lead Contact
*Correspondence: bruno.monier@univ-tlse3.fr (B.M.), magali.suzanne@univ-tlse3.fr (M.S.)
https://doi.org/10.1016/j.devcel.2019.05.027

SUMMARY

Mechanical forces are critical regulators of cell shape changes and developmental morphogenetic processes. Forces generated along the epithelium apico-basal cell axis have recently emerged as essential for tissue remodeling in three dimensions. Yet the cellular machinery underlying those orthogonal forces remains poorly described. We found that during *Drosophila* leg folding cells eventually committed to die produce apico-basal forces through the formation of a dynamic actomyosin contractile tether connecting the apical surface to a basally relocalized nucleus. We show that the nucleus is anchored to basal adhesions by a basal F-actin network and constitutes an essential component of the force-producing machinery. Finally, we demonstrate force transmission to the apical surface and the basal nucleus by laser ablation. Thus, this work reveals that the nucleus, in addition to its role in genome protection, actively participates in mechanical force production and connects the contractile actomyosin cytoskeleton to basal adhesions.

INTRODUCTION

Animal development relies on the dynamic remodeling of tissues to create germ layers. Subsequently, specialized organs are formed and sculpted into stereotyped shapes, a prerequisite for their function. Invagination or bending of epithelial sheets is essential to convert an initially flat polarized epithelia into complex three-dimensional structures during organogenesis (Davidson, 2012; Pearl et al., 2017). For example, during gastrulation, it allows to segregate tissues with distinct fate (Keller et al., 2003). Another example is neural tube formation in vertebrates, which relies on local bending and closure of the neural plate, and failure to correctly invaginate may lead to developmental defects such as spina bifida or anencephaly associated with defective neural tube formation (Colas and Schoenwolf, 2001).

Acquisition of a new shape at the tissue scale is triggered by the coordinated change in shape of individual cells. Apical constriction of epithelial cells is usually associated with tissue invagination and is thought to trigger tissue remodeling (Martin and Goldstein, 2014). Cell shape changes essentially rely on the intrinsic property of cells to generate forces. At the subcellular scale, mechanical forces are usually produced by association of the molecular motor non-muscle Myosin II with filamentous actin (Lecuit et al., 2011). Mechanical forces, generated in the plane of adherens junction, represent an important driving force in epithelial remodeling (Chanet and Martin, 2014; Heisenberg and Bellaïche, 2013; Umetsu and Kuranaga, 2017). Much of our understanding comes from the fine characterization of the early steps of *Drosophila* mesoderm invagination. In this case, a pulsatile actomyosin network accumulates medio-apically in invaginating cells (Martin et al., 2009; Mason et al., 2013). The actomyosin meshwork organizes radially, with F-actin sarcomere-like cables emanating from the cell center (Coravos and Martin, 2016). A molecular clutch allows coupling with E-cadherin-catenin complexes (Roh-Johnson et al., 2012). Transient contraction of the actomyosin network then causes a decrease in apical surface area that is stabilized by a ratchet mechanism while forces are transmitted to neighbors through adherens junctions (Martin et al., 2009, 2010). Additional mechanisms, such as junctional rather than medio-apical accumulation of actomyosin, also enable apico-basal force generation (Hildebrand, 2005; Owaribe et al., 1981).

Although apical constriction plays a key role in tissue folding, recent evidence points at a more complex situation. Indeed, formation of salivary glands in *Drosophila*, which originate from the local invagination of an epithelial placode, has recently been shown to proceed when apical constriction is inhibited (Chung et al., 2017). Such an observation highlights the fact that additional or redundant mechanisms cooperate with apical constriction to promote efficient and stereotyped tissue invagination, at least in some contexts.

Interestingly, orthogonal apico-basal forces represent critical input with regard to tissue remodeling from two to three dimensions. (Kondo and Hayashi, 2015; Monier et al., 2015; Pearl et al., 2017; Sherrard et al., 2010; Yang et al., 2017). Indeed, during ascidian gastrulation, endoderm invagination is a two-step process beginning with Rok-dependent apical actomyosin contraction leading to apical constriction. This step is followed by basolateral myosin accumulation and cell shortening. Importantly, specific blocking of apical constriction and not cell shortening does not prevent tissue invagination, although folding is less pronounced (Sherrard et al., 2010). In the distal part of the *Drosophila* leg epithelium, the appearance of folds relies on localized apoptosis (i.e., programmed cell death) (Manjón et al., 2007). A combination of *in vivo* genetic manipulations

This is an open access article under the CC BY license (http://creativecommons.org/licenses/by/4.0/).

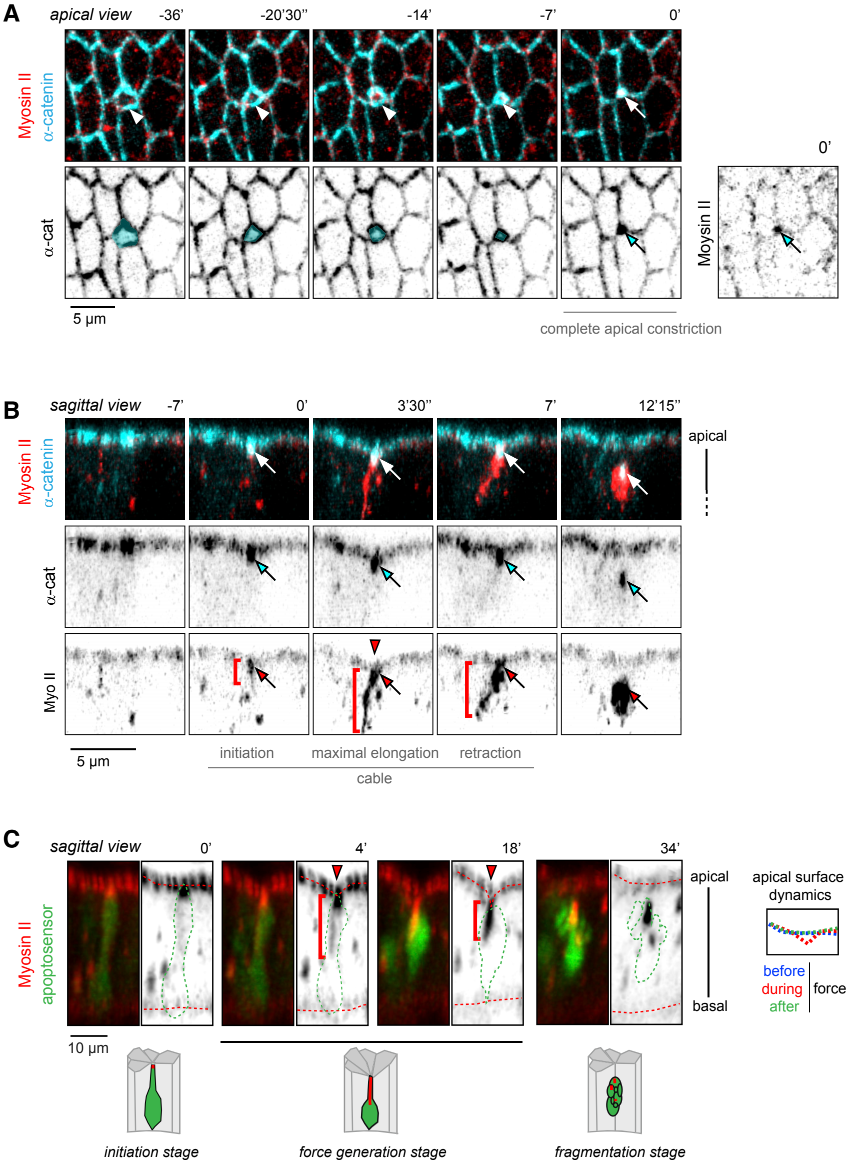

Figure 1. Coordinated Dynamics of Adherens Junction and Myosin II in Apoptotic Cells

(A and B) Stills from a movie showing apical (A) and sagittal views (B) of the same cell stained with the adherens junction marker α-catenin (cyan) and myosin (red) (n = 11).

(A) White arrowheads on the merge images show apoptotic cell apical constriction until the apex is completely closed. Apoptotic cell is colored in cyan on the black and white images. Myosin starts to accumulate at adherens junctions when they are closed (white and cyan arrows).

(B) White, cyan, and red arrows show the colocalization between Myosin II and α-catenin during the apoptotic process. The red arrowhead points at the transient deformation of the apical surface of the epithelium triggered by the apico-basal force. The red brackets follow the myosin cable.

(C) Stills form a movie showing the myosin cable (red) inside the apoptotic cell (green) (n = 15) and the corresponding schemes. The red brackets follow the myosin cable whose maximal extension reaches the middle of the cell (4′). The apoptotic cell is outlined in green and the apical and basal surfaces are highlighted by red dotted lines on the black and white images. The red arrowhead points at the transient deformation of the apical surface. The panel on the right illustrates apical surface dynamics (color-coded) during apoptosis.

and *in silico* modeling showed that an apico-basal pulling force is required in dying cells to trigger folding of surrounding living cells (Monier et al., 2015). This force relies on an elongated actomyosin structure that forms along the apico-basal cell axis of dying cells. How this actomyosin structure is tethered to specific cellular components in order to create a force at the cell scale remain unknown (Kiehart, 2015; Monier et al., 2015). Such reports demonstrate the importance of apico-basal forces complementing apical constriction to produce highly stereotyped tissue invagination. However, no clear mechanism was reported regarding how cells reorganize to produce efficient forces along their apico-basal axis.

In order to identify the underlying mechanism of orthogonal cellular force production, we focused on the folding process of the *Drosophila* developing leg. We show that apoptotic cells relocate their nucleus at the vicinity of basal adhesions and trap it in an F-actin meshwork that restrains nucleus movement. Further, the contractile actomyosin cable-like structure links adherens junction to the stabilized basal nucleus. This transient contractile connection between adherens junctions and basal adhesions via the nucleus generates a force at the cellular scale that modifies the shape of the epithelium.

RESULTS

Apoptotic Apical Myosin II Cable Dynamics

We previously reported that during apoptotic force generation, Myosin II creates an apico-basal accumulation emanating from the apical region of the cell (Monier et al., 2015), hereafter named "apico-basal myosin cable". Force generation at the cell scale necessitates that the intracellular molecular force-producing machinery is linked to stable anchoring points to transmit forces to neighboring cells. To identify the localization of the various anchoring points of the apico-basal myosin cable, we first characterized more precisely Myosin II dynamics following myosin live by performing time-lapse imaging with co-labeling of the component of adherens junctions, α-catenin. Constriction of the apical surface is the first step of dying cell remodeling (Kuipers et al., 2014; Lubkov and Bar-Sagi, 2014; Rosenblatt et al., 2001; Schott et al., 2017). Myosin recruitment starts at the end of apical constriction where α-catenin accumulates (Figures 1A and 1B; Video S1). Then, the Myosin II cable forms, extends basally to a maximum, then retracts, and eventually detaches from the apical surface concomitantly with apoptotic adherens junctions internalization

CellPress

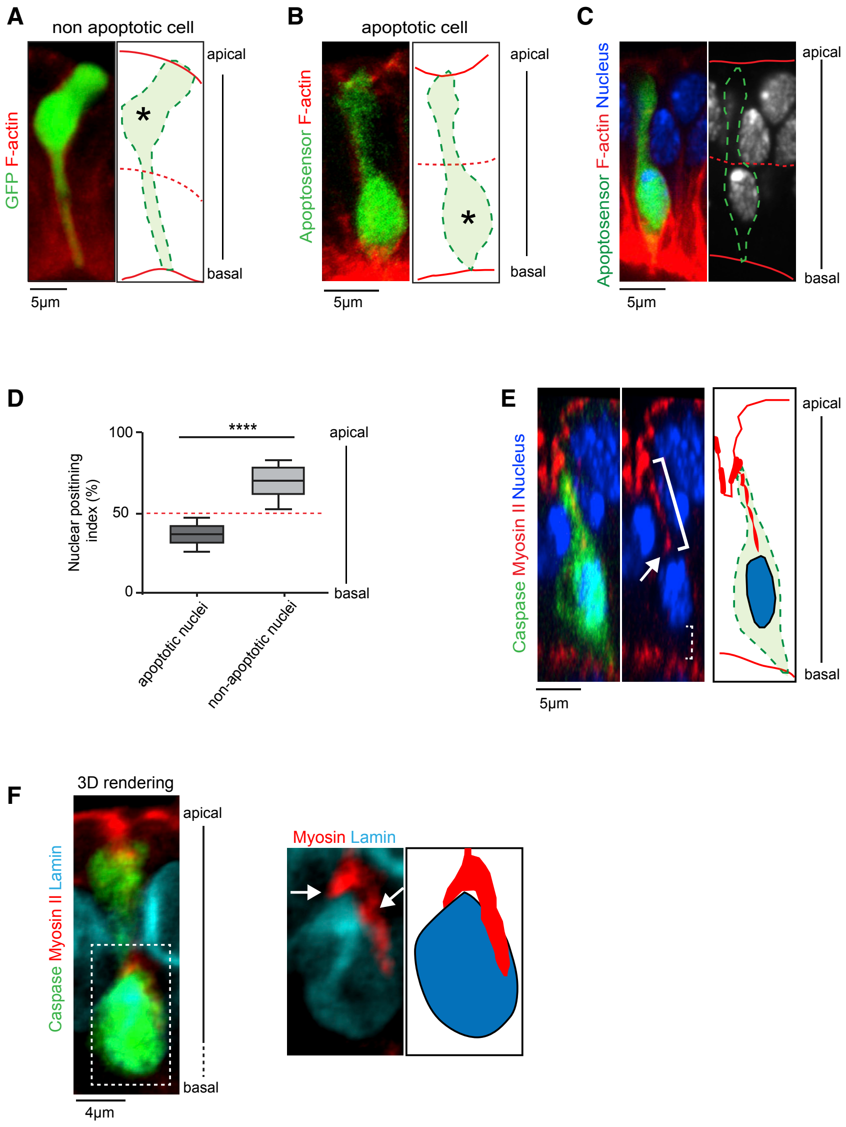

Figure 2. Proximity between the Myosin Cable and the Basal Apoptotic Nucleus

(A and B) Sagittal views and schematic representations showing the localization of cell bodies (asterisk) in individual nonapoptotic (A) and apoptotic (B) cells (n = 24 and 23). Cells are outlined in dotted green lines in the schematic representations.

(C) Sagittal view showing nuclei positioning (blue or white) in apoptotic (green or dashed green line) and nonapoptotic cells. Only the apoptotic nucleus is located in the basal half of the epithelium. In (A–C), apical and basal surfaces are schematized by a red line and the midplane of the epithelium by a red dashed line.

(D) Box plot representation of the quantification of nuclei localization along the apico-basal cell axis in apoptotic (n = 25) and nonapoptotic (n = 115) cells. Wilcoxon statistical test: p value < 0.0001 (****). The midplane of the epithelium is indicated by a dashed red line.

(E) Sagittal view and schematic representation showing the myosin cable (highlighted by the white bracket) which extends toward the basal apoptotic nucleus (arrow) (n = 13). No myosin accumulation can be detected between the apoptotic nucleus and the basal-most region of the cell (dashed bracket).

(F) (Left) 3D reconstructions of a general view of an apoptotic cell (green) and a close-up of the nucleus (Lamin-TagRFPt in cyan) (n = 9). (Right) Schematic representation. The myosin cable (red) contacts the apical side of the nucleus (white arrows).

(E and F) Apoptotic cells are stained in green with an anti-cleaved Dcp1 antibody.

See also Figure S1.

(Figure 1B; Video S1). Importantly, Myosin II appears tightly connected to adherens junctions throughout the whole apoptotic process, from myosin cable initiation to maximal cable extension to cable retraction. Hence, like during other invagination processes (Martin et al., 2010; Roh-Johnson et al., 2012), analysis of Myosin II dynamics suggests that the apico-basal myosin cable is anchored to adherens junctions during the whole process of apico-basal force generation.

The Apical Myosin II Cable Targets the Basally Relocated Nucleus

The linear organization of the Myosin II apoptotic structure suggests the presence of a second anchoring point at the basal end of the myosin cable, i.e., opposite the adherens junctions. To identify this potential anchoring point, we next performed live imaging by coexpressing Myosin II and a fluorescent reporter of caspase activity that highlights the cytoplasm of apoptotic cells (Schott et al., 2017). We found that at maximal extension the apico-basal myosin cable ends in the middle of the cell (Figure 1C). The apico-basal force, which transiently and locally deforms the apical surface of the epithelium, is initiated at this stage. Then, the cable shortens and eventually detaches from the apical surface at the time of apoptotic cell fragmentation (Figure 1C).

The basal anchoring point of the myosin cable could exist in both apoptotic and nonapoptotic cells or, alternatively, be a specialized structure created when cells turn on the apoptotic program in order to provide basal resistance to the apico-basal myosin cable. To discriminate between those hypotheses, we labeled individual nonapoptotic epithelial cells and compared their morphology with apoptotic ones. Interestingly, we found that, in the leg pseudostratified epithelium, apoptotic cells adopt a shape drastically different from their nonapoptotic neighbors. Indeed, nonapoptotic cells present a large cell body located in the apical half of the epithelium and connected to the basal surface by a long, thin extension (Figure 2A). On the contrary, apoptotic cells that have not entered the fragmentation stage possess a narrow apical section and a large cell body localized basally (Figure 2B). As the nucleus is the cell's biggest organelle, we wondered whether its positioning could account for the specific shape of dying cells. Indeed, we found that while nuclei of nonapoptotic cells are located on the apical half of epithelial cells, nuclei of apoptotic cells are systematically located on the basal half (Figures 2C and 2D). At that stage of apoptosis, the nucleus is still

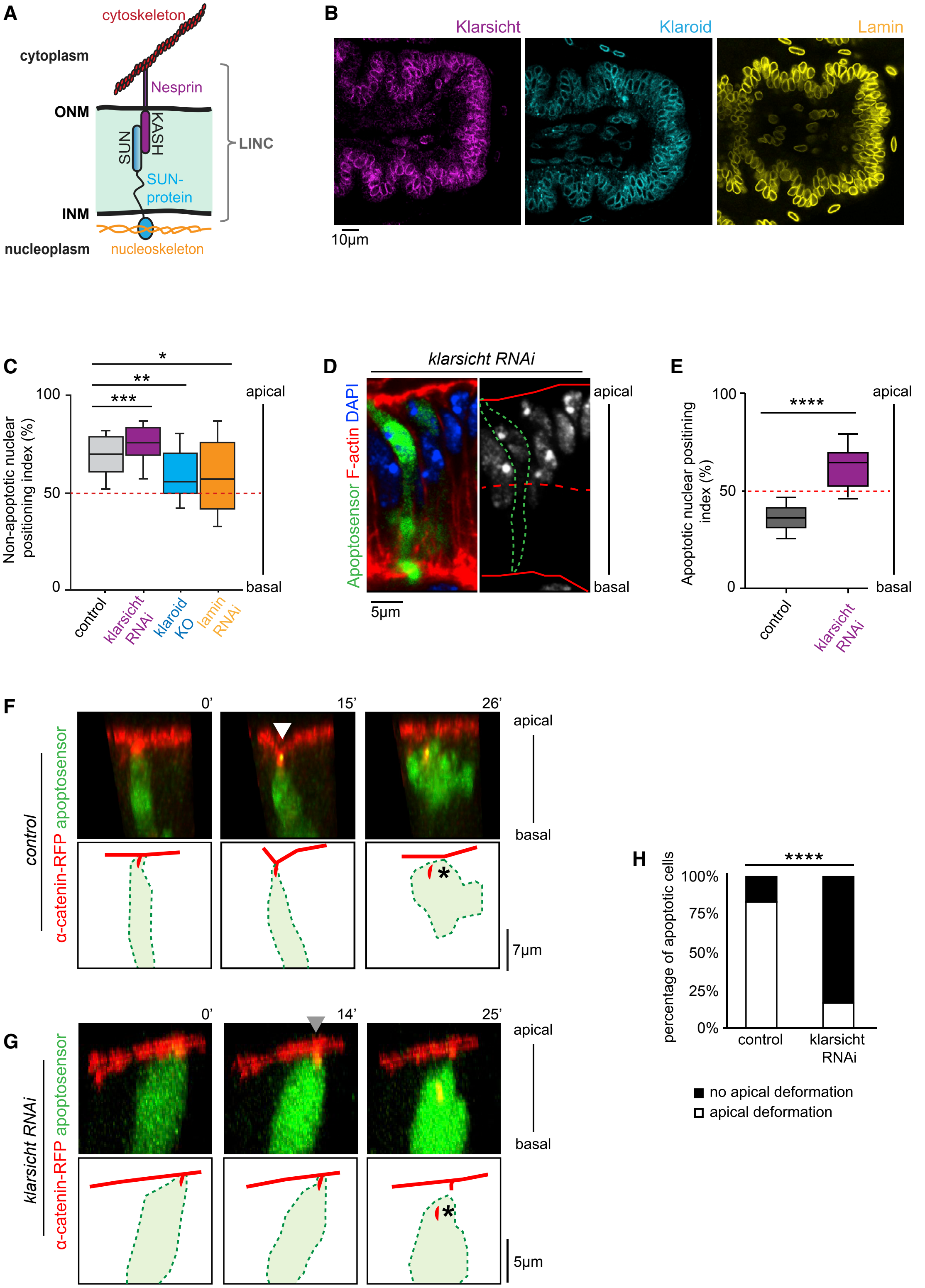

Figure 3. Basal Positioning of the Nucleus Is Essential for Apico-basal Force Generation

(A) Scheme describing the LINC complex made of SUN- and KASH-domain proteins. The color-code for Nesprin, SUN-protein, and nucleoskeleton is used in (B) and (C). ONM, outer nuclear membrane; INM, inner nuclear membrane.

(B) Perinuclear localization of LINC members (Klarsicht and Klaroid) and Lamin in the leg tissue.

(legend continued on next page)

morphologically similar to nonapoptotic nuclei, although we observe a reduction of Lamin levels (using a Lamin-TagRFPt endogenous fusion protein), which may render the apoptotic nucleus softer or modulate its interaction with the cytoskeleton (Figures S1A and S1B).

To test if the apoptotic nucleus could be part of the apico-basal force machinery, we investigated whether the apical Myosin II cable targets the nucleus. We observed that the basal end of the actomyosin cable is in close vicinity of the apoptotic nucleus (Figure 2E). Moreover, 3D reconstruction images of the nucleus periphery labeled with Lamin-TagRFPt showed that the basal end of the cable runs along the apical side of the nucleus (Figure 2F). These observations suggest a physical association between the apico-basal Myosin II cable and the nucleus.

Basal Nucleus Relocation Is Essential for Apico-basal Force Generation

To test whether the nucleus is required for apico-basal force generation, we looked for a way to inhibit the basal relocation of the apoptotic nucleus. One candidate was the linker of nucleoskeleton and cytoskeleton (LINC) complex, a macromolecular complex involved in nucleus localization in a number of cellular contexts ranging from fibroblast migration to interkinetic nuclear movement in the vertebrate central nervous system (Gundersen and Worman, 2013; Lee and Burke, 2018). The LINC is composed of Nesprins spanning the external nuclear envelope interacting with SUN-domain proteins spanning the internal nuclear envelope. Nesprins also interact with the cytoskeleton while SUN proteins bind to Lamin within the nucleus (Figure 3A). In the *Drosophila* leg, we observed perinuclear localization for the sole somatic SUN-protein, Klaroid, similarly to Lamin (Figure 3B). Among the two fly Nesprins, Klarsicht has also a specific perinuclear localization (Figures 3B and S2).

We next tested whether inactivation of those LINC components affects nucleus positioning in this tissue, in parallel to Lamin inactivation. We found that inactivation of *klaroid* and *lamin* leads to nonapoptotic nuclei mispositioning, with most nuclei found in the middle plane of the leg epithelium instead of being located in the apical half (Figure 3C). This indicates a general function of *klaroid* and *lamin* in nuclei positioning in the developing leg. On the contrary, *klarsicht* inactivation had a very limited impact on the position of nonapoptotic nuclei, which remained apical (Figures 3C and 3D). However, we observed that *klarsicht* inactivation blocks apoptotic nuclei relocation as shown by the presence of all apoptotic nuclei in the apical half of the epithelium (Figures 3D and 3E, compare with Figure 2C).

We choose to focus our analysis on the *klarsicht* loss-of-function background. Apoptotic cells exert a force that transiently deforms the apical surface of the epithelium (Monier et al., 2015) (Figure 3F; Video S2). Therefore, to directly address whether the nucleus contributes to apico-basal force generation, we followed the shape of the apical surface by time-lapse imaging in a context of *klarsicht* inactivation. Importantly, we found that, in this context where the nucleus remains apical, most apoptotic cells are unable to transiently deform the apical surface of the epithelium (Figures 3G and 3H; Video S3). This reveals that apico-basal forces are considerably reduced or absent in this context, strongly suggesting that nucleus positioning is critical for apoptotic force generation.

Basal Anchoring of the Apoptotic Nucleus by an Actin Network Linked to Basal Adhesion

To act as the basal Myosin II cable anchoring point, the apoptotic nucleus would have to resist the intracellular force developed by the actomyosin cytoskeleton. For this, it might either be stabilized basally or, being the cell's largest organelle, it might be able to offer resistance by itself. To discriminate between these hypotheses, we used high temporal resolution imaging to compare the movement of nonapoptotic nuclei and basal apoptotic nuclei during the period preceding cable formation (i.e., before the force-generation stage). Interestingly, nuclear tracking showed that apoptotic nuclei are less mobile than nuclei of living neighboring cells (Figure 4D, compare DMSO conditions). Because at that stage of apoptosis the nucleus is morphologically similar to the nucleus of nonapoptotic cells (Figure S1A), the difference in nuclei velocity suggests the presence of a stabilizing mechanism specific for apoptotic nuclei.

We next sought to characterize how the nucleus is stabilized in apoptotic cells. Although the LINC complex participates in nucleus anchoring in several contexts (Tapley and Starr, 2013), its involvement in apoptotic nucleus relocation precludes analysis of its possible role in basal nucleus stabilization. We thus focused on cytoskeleton components. In the leg epithelium, microtubules form an apical cap and are enriched along lateral membranes in the apical half of the cell, with limited or no localization on the basal side. By contrast, F-actin strongly accumulates in apical and basal domains, with weaker staining along lateral membranes (Figures 4A–4C). The relative distribution of those two cytoskeletal networks prompted us to focus on the F-actin cytoskeleton, as it is more likely to be in contact with basally relocated nuclei. By performing high-resolution microscopy on early apoptotic cells (prior to the myosin cable or

(C) Box plot of the apico-basal distribution of nonapoptotic nuclei in the epithelium of control (n = 115), *klarsicht* RNAi (n = 67), *lamin* RNAi (n = 24), and *klaroid* mutant (n = 21). The red dotted line indicates the midplane of the epithelium in the graph. Mann & Whitney test: p value < 0.0001 for ***, <0.0024 for **, and <0.0144 for *.

(D) Sagittal views showing that the apoptotic nucleus is located apically in a *klarsicht* RNAi context. Nuclei are in blue or white and the apoptotic cell is indicated in green or with a green dotted line. Apical and basal surfaces of the epithelium are outlined in red and the midplane of the epithelium with a red dotted line.

(E) Box plot of apoptotic nucleus distribution in the apico-basal cell axis in control (n = 25) and *klarsicht* RNAi (n = 33) contexts. The red dotted line indicates the midplane of the epithelium in the graph. Mann & Whitney test: p value < 0.0001 (****).

(F and G) Stills from time-lapse movies and corresponding schemes showing apoptotic cell dynamics in control (F) and *klarsicht* RNAi (G) contexts. Arrowheads mark the presence (F, in white) or the absence (G, in gray) of deformation of the apical surface. Black stars indicate the adherens junction apical accumulation detachment. t = 0′ is set at the end of the apoptotic apical constriction.

(H) Histogram showing the proportion of apoptotic cells deforming or not the apical surface of the epithelium in control (n = 23) and *klarsicht* RNAi (n = 12) contexts. Fisher statistical test: p value < 0.0003 for ****.

See also Figure S2.

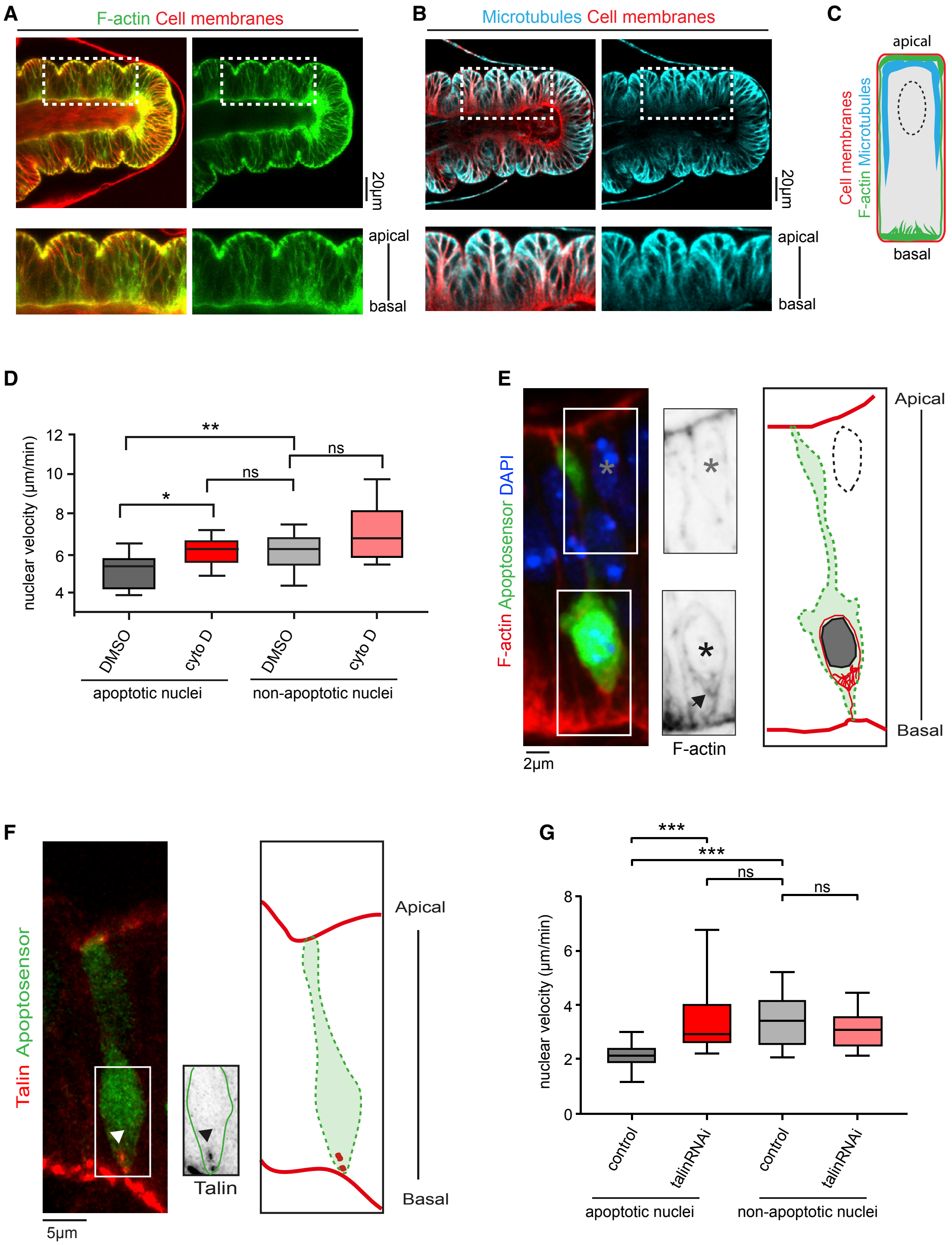

Figure 4. A Basal Adhesion-F-Actin Network Stabilizes the Basal Apoptotic Nucleus

(A and B) Confocal images showing distribution of F-actin (Utrophin-GFP reporter, green, A) and microtubules (MAP205::GFP protein trap, cyan, B) in live leg discs. The cell membranes are stained in red using the lipid dye FM4-64 to visualize the leg epithelium.

(C) Schematization of F-actin and microtubules distribution in the leg disc observed in (A) and (B). F-actin is enriched in the cell's apical and basal domains and, to a lesser extent, in the lateral domain. Microtubules accumulate mainly in apical and lateral domains. They are essentially absent from the cell's basal pole.

(D) Box plot representation of apoptotic and nonapoptotic nuclei displacement per minute in control (DMSO; n = 9 and 9) and after treatment by Cytochalasin D (n = 21 and 21). Wilcoxon statistical test: p value < 0.0039 for ** and Mann Whitney statistical test: p value < 0.0246 for * and nonsignificant for n.s.

(E) Sagittal view and scheme showing the presence of a basal F-actin structure (black arrow) contacting the apoptotic nucleus (black star), but not a nonapoptotic nucleus (gray star). F-actin is in red (left) or black (right), nuclei in blue, and the apoptotic cell in green (n = 24).

(legend continued on next page)

force-generation stage), we identified strong F-actin accumulation running between the basal apoptotic nucleus and the most basal part of the cell, while no specific F-actin accumulation is detected in close vicinity to nonapoptotic nuclei (Figure 4E). This observation suggests that relocation of the nucleus in the early apoptotic phase could bring it in close vicinity with an F-actin basal network, likely poorly contractile since no Myosin II is detected basally (Figure 2E).

To determine whether the actin cytoskeleton anchors the nucleus basally, we performed tracking of apoptotic nuclei following actin destabilization. We set up conditions in which short incubation of leg discs with Cytochalasin D-containing culture medium causes a substantial decrease in polymerized actin yet does not lead to altered tissue morphology (not shown). In this condition, dynamics of nonapoptotic nuclei is not significantly affected (Figure 4D, compare nonapoptotic nuclei in DMSO and Cytochalasin D conditions). On the contrary, apoptotic nuclei mobility was increased after actin destabilization, reaching the velocity of nonapoptotic nuclei (Figure 4D, compare apoptotic nuclei in Cytochalasin D condition, with nuclei in DMSO). These results indicate that an F-actin network specifically stabilizes the apoptotic nucleus.

Actin-dependent stabilization of the nucleus most probably necessitates a basal anchor at the membrane to provide resistance. Although dying cells are commonly viewed as detaching from the matrix, we found that basal adhesions are maintained during early apoptotic stages (Figure 4F), as are apical adhesions (Lubkov and Bar-Sagi, 2014; Monier et al., 2015). This suggests that basal adhesions might act as the ultimate basal anchoring point of the system. To directly assess this idea, we used RNAi to inactivate Talin, a key component of focal adhesion that mechanically links integrin receptors to the actin cytoskeleton (Klapholz and Brown, 2017). In this context, although Talin level is greatly reduced, cellular integrity is unaffected (Figure S3). We therefore compared nuclei stability in control and Talin RNAi conditions. The dynamics of apical nonapoptotic nuclei, which are located far away from basal adhesions, were unaffected in Talin RNAi condition. However, the velocity of apoptotic nuclei was drastically increased compared to control apoptotic nuclei, becoming similar to the velocity of nonapoptotic nuclei (Figure 4G). Therefore, apoptotic nuclei behave similarly either when F-actin is destabilized or when Talin function is reduced. Altogether, those results point toward a model in which a basal F-actin cytoskeleton network linked to basal adhesion stabilizes the apoptotic nucleus prior to the force-generation stage.

Anchoring the Nucleus Basally Provides Resistance Required for the Apico-basal Force

We next focused on the force-generation stage and asked if the stabilizing mechanism of apoptotic nuclei identified above could be involved in force generation. We therefore followed the dynamics of both Myosin II and the nucleus during the apoptotic process, both in a control and in a context in which basal anchoring is deficient.

In the control, we observed that the apoptotic nucleus is located basally before cable formation. Then, the cable extends from the apical surface of the epithelium to the upper part of the nucleus. Finally, the nucleus progressively moves apically, remaining in close vicinity to the Myosin II cable as it shortens (Figures 5A and S4; Video S4). Interestingly, the nucleus movement is composed of two upward phases separated by a transient break shown by slower and random motion, suggesting a physical resistance halfway up (Figure 5C).

Since F-actin depolymerization would alter not only nucleus stabilization but also apical contractile cable formation (Monier et al., 2015), we tested the role of nucleus basal stabilization by inactivating Talin. We show that although Talin plays a critical role in limiting basal apoptotic nuclear movement, it has no impact on apoptotic nucleus basal relocation or apico-basal myosin cable formation, thus allowing us to follow nucleus dynamics during the whole apoptotic process (Figure 5B; Video S5). In this context, although the nucleus moves apically as observed in the control, quantitative analysis reveals that nucleus dynamics is altered. Indeed, in absence of basal anchoring, upward movement of the nucleus is rather regular, with no or limited break in upward nuclear motion (Figures 5D and 5E). Hence, apico-basal myosin cable and nucleus dynamics are correlated in both contexts, although upward movement is continuous when basal anchoring is defective in *talin* loss of function. These data suggest that, at the force-generation stage, the Myosin II cable is anchored to the nucleus, which resists to the force thanks to the basal F-actin-basal adhesion network.

To directly test the importance of nucleus basal anchoring in providing resistance to the apico-basal myosin cable contraction, we inactivated *talin* and analyzed the ability of the apoptotic cells to deform the apical surface. Importantly, we found that most apoptotic cells devoid of Talin are no longer able to trigger the apical surface deformation of the surrounding epithelium (Figures 5F and 5G; Video S6 compare with Figure 3F and Video S2). This indicates that the apoptotic force is either abolished or strongly reduced when basal adhesions are weakened. Therefore, basal adhesions are critical to stabilize the nucleus and offer effective resistance to Myosin II contraction during apico-basal force generation.

Transmission Path of the Apico-basal Force

Combined, our results point toward a model in which the myosin cable would contract and generate forces that would be transmitted, on the one hand, toward the apical surface and, on the other hand, toward the basal resisting apoptotic nucleus. To strengthen our understanding of the apico-basal force transmission path, we therefore set up laser ablation experiments. Based

(F) Sagittal view and scheme showing the presence of Talin (in red and black, arrowheads) in an apoptotic cell (green) (n = 4).

(G) Box plot representation of apoptotic and nonapoptotic nuclei displacement per minute in control (n = 12 and 11) and *talin* RNAi (n = 11 and 10). Wilcoxon statistical test: p value < 0.001 for ***, and Mann Whitney statistical test: p value < 0.0007 for ***, and nonsignificant for n.s. (D and G) A non-paired Mann Whitney test was used to compare nuclei displacement between conditions, except for comparison of apoptotic versus nonapoptotic nuclei in DMSO (D) or control (G) for which we used a paired Wilcoxon test.

In (D–G), apoptotic cells are at the initiation stage, before the generation of the apico-basal force.

See also Figure S3.

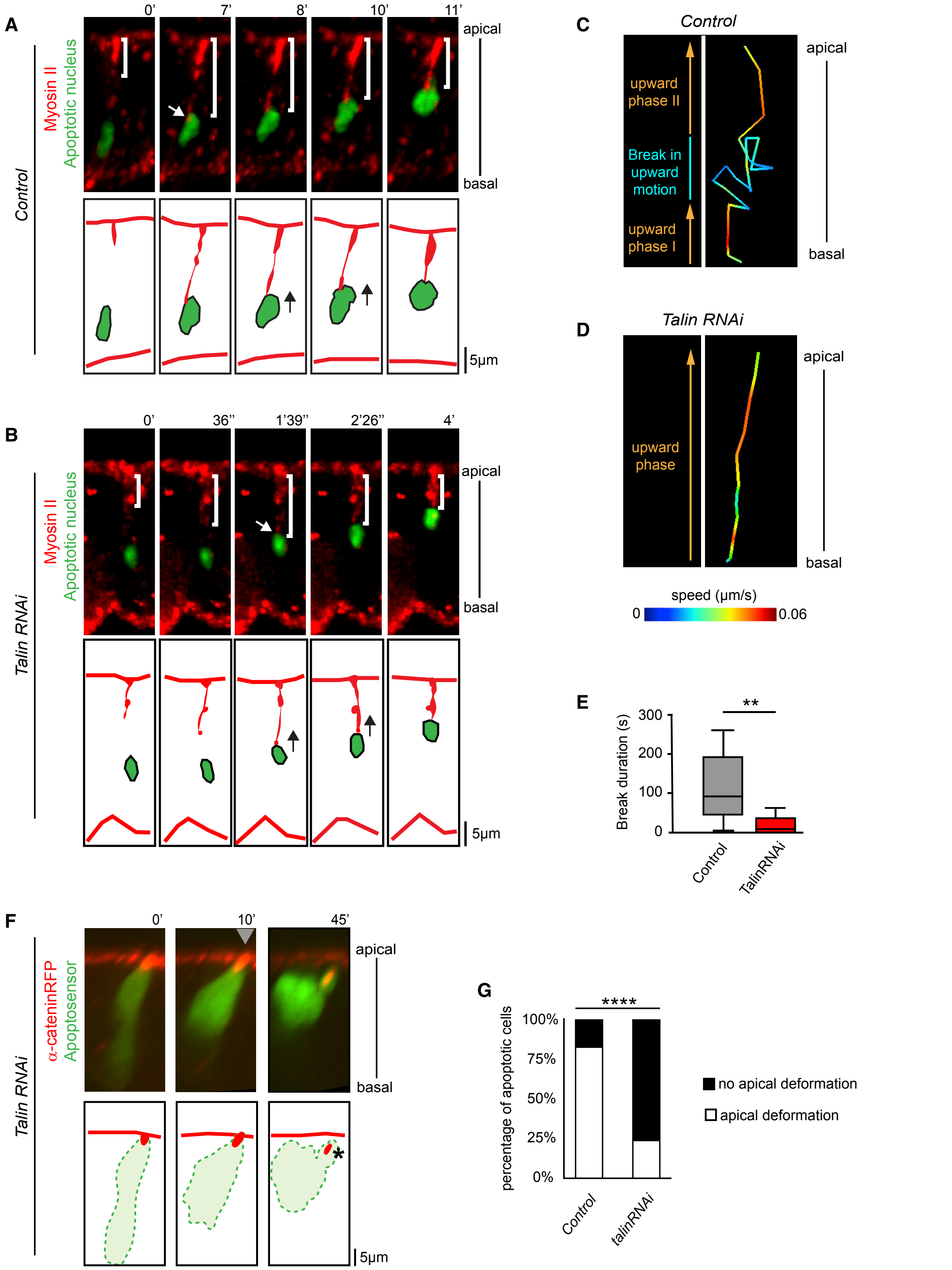

(legend on next page)

on the recognition of apoptotic cells by the presence of Myosin-GFP cables running from adherens junctions to the middle of the epithelium in the prospective fold domain, we found that cuts in the middle of myosin cables lead to rapid retraction of the opposite ends of the cables (Figures 6A, 6B, and 6E). We further notice a recoil of the apical surface after ablation of the apoptotic cable (Figures 6B and 6F). On the contrary, apico-basal laser cut in nonapoptotic cells leads to weak or even absence of retraction and has no effect on apical surface (Figures 6C–6F). These results show that the myosin cable formed in apoptotic cells is under tension and that the myosin cable-dependent force is transmitted toward the apical region of the dying cell and subsequently to the neighbors.

We further investigated whether apico-basal forces are also transmitted to the basal apoptotic nucleus. For this purpose, we used the recently developed apoptosensor, DBS-S (Baena-Lopez et al., 2018). DBS-S is a membrane-bound GFP that relocalizes to the nucleus upon initiator caspase activation. Upon UV induction in the larval wing disc, basal apoptotic nuclei become fluorescent and move apically (Baena-Lopez et al., 2018) in a process reminiscent of nuclear dynamics of endogenously dying cells in prepupal legs (Figure 5A). We therefore characterized nucleus behavior upon cable ablation by combining DBS-S to myosin-TagRFPt. In most cases, the nucleus moves basally following laser ablation (Figures 6G and 6H), supporting the notion of force transmission to the nucleus. The absence of nuclear movement in the remaining cases might be due to ablation performed just before force generation, the stage during which nucleus upward movement is initiated. To further confirm that myosin cable-dependent forces are transmitted to the nucleus, we followed the dynamics of the nuclear envelope overtime. Interestingly, while the apoptotic nuclear envelope is initially round, it deforms and adopts a pointed shape during nuclear upward movement (Figures 6I and 6J). This is consistent with the observation of Myosin II running around the apical surface of the nucleus (Figure 2F). On the contrary, the envelope of nonapoptotic cells remains round over the same timescale (Figure 6J). Altogether, we propose that apico-basal forces are transmitted both apically and basally during the apoptotic process.

Perturbation of Apico-basal Force Generation Affects Fold Formation

We previously reported that apoptosis is necessary for the progressive formation of folds in leg tarsal segments. Moreover, artificial induction of apoptosis is sufficient to create ectopic folding in the wing disc. This ectopic folding relies on apoptotic forces since inhibition of myosin activity specifically in dying cells prevents the formation of ectopic folds (Monier et al., 2015). We therefore reasoned that tempering with components of the cellular machinery of apoptotic apico-basal force generation should compromise fold formation in the leg. We focused on the fold that separates tarsal segments 4 and 5 (T4/5) because, in this domain, folding starts at the beginning of metamorphosis. This allowed us to check that epithelium shape is normal prior to the onset of fold formation in absence of Talin or Klarsicht (Figure S3; data not shown). In both RNAi contexts, we observed shallow or absent T4/5 folds in most of the legs analyzed, at a stage where deep folds are formed in control legs (Figures 7A and 7B). These results demonstrate that apico-basal forces created by apoptotic cells are critical for correct fold formation in the developing leg.

DISCUSSION

A Cellular Reorganization That Creates Apico-basal Forces: Lessons from Dying Cells

A growing body of evidence support the notion that apical constriction cooperates with additional forces to drive tissue invagination. During mesoderm invagination in flies, very early steps of the process that lead to formation of a cup-shape need apical constriction (Martin et al., 2009) but are unlikely to necessitate an apico-basal force since this stage can be reached in acellular embryos that lack lateral membranes (He et al., 2014). However, at later, less understood stages of mesoderm invagination, an increase in apico-basal tension has been inferred by video-force microscopy (Brodland et al., 2010). This is consistent with *in silico* modeling that shows that apical and apico-basal forces must cooperate to produce an efficient and stereotyped pattern of invagination (Monier et al., 2015). This view is supported by work that shows that endoderm invagination in ascidian embryos is a two-step process in which blocking apical constriction does not prevent invagination driven by apico-basal forces (Sherrard et al., 2010). However, the cellular machinery involved in the generation of such apico-basal forces remains to be identified.

We focused on apoptotic cells for several reasons: (1) apoptotic cells generate apico-basal forces that trigger invagination in the *Drosophila* leg (Monier et al., 2015), (2) individual apoptotic cells can be followed *in vivo* thanks to the recent

Figure 5. Basal Apoptotic Nucleus Stabilization Is Essential for Efficient Apico-basal Force Generation

(A and B) Stills from time-lapse movies and schematic representations showing the dynamics of Myosin II (red) and the apoptotic nucleus (green) after post-acquisition treatment (see Figure S4 and STAR Methods for details) in control (A, n = 12) and *talin* RNAi context (B, n = 10). White brackets highlight the dynamics of apoptotic myosin cable, white arrows indicate cable-nucleus contact, and black arrows on the schemes indicate nuclei movements.

(C and D) Comparison of apoptotic nuclei upward movements in control (C, n = 12) and *talin* RNAi (D, n = 10) contexts. Trajectories are color-coded to reveal nuclei speed.

(E) Box plot representation of the duration of the break in nucleus upward movement in control and *talin* RNAi contexts based on the movement observed in (C) and (D). Mann Whitney test: p value < 0.0039 for **.

(F) Still from a time-lapse movie and corresponding schemes showing apoptotic cell dynamics in *talin* RNAi context. No apical surface deformation, indicative of an absence of apico-basal force, is observed (gray arrowhead). Adherens junction apical accumulation detaches normally (star). t = 0′ is set at the end of the apoptotic apical constriction (n = 23).

(G) Histogram showing the proportion of apoptotic cells deforming or not the apical surface of the epithelium in control (n = 23) and in *talin* RNAi (n = 25). Fisher statistical test: p value < 0.0001 for ****.

See also Figure S4.

CellPress

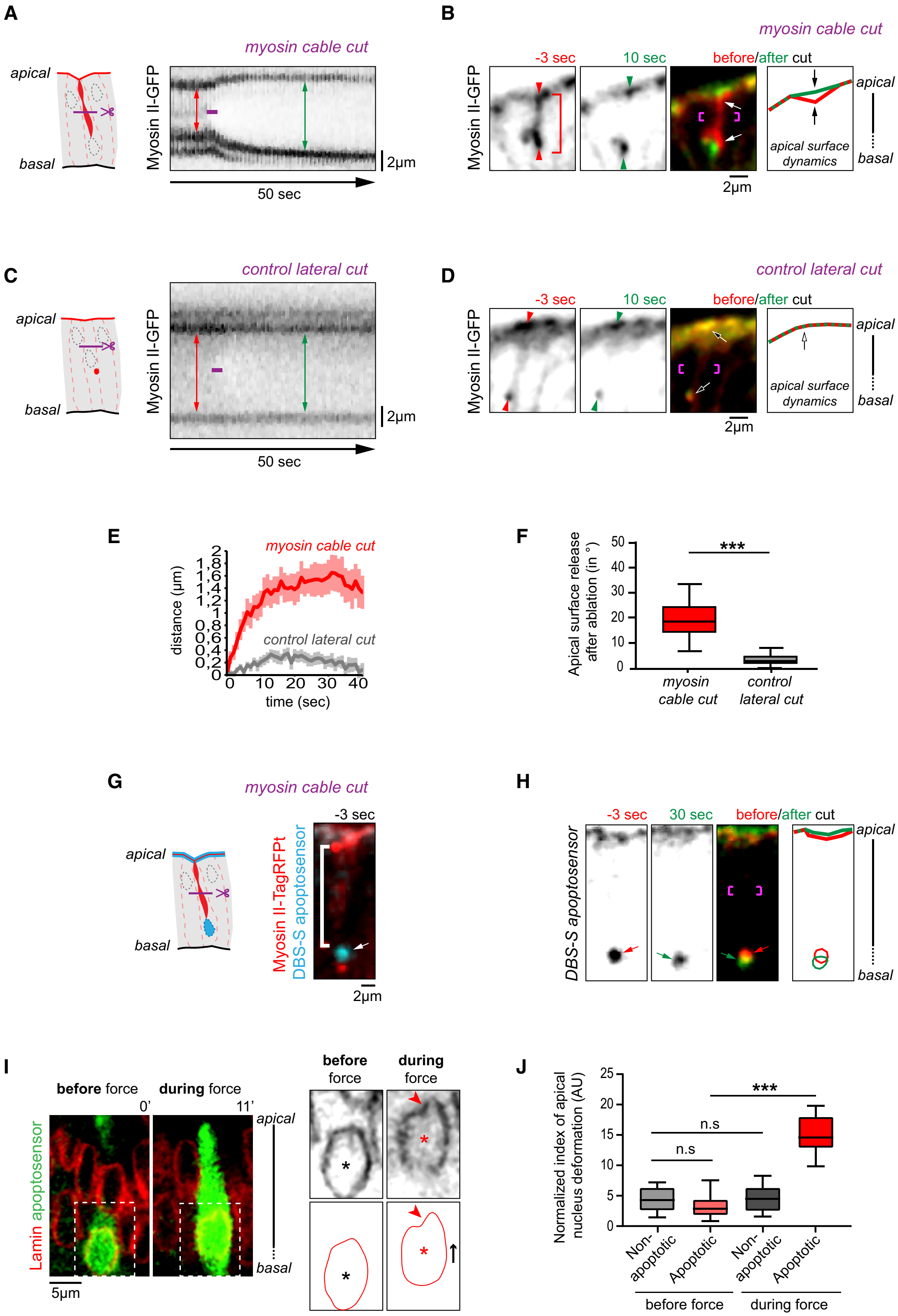

Figure 6. Forces Generated by the Apoptotic Myosin Cable Are Transmitted to the Apical Surface and the Nucleus

(A–D) Laser ablation of apico-basal myosin cable (A and B) and control lateral cut in nonapoptotic cells (C and D). In kymographs, red and green double arrows indicate the distance between two dots before and after cut respectively. Cuts were performed at the level of the apico-basal myosin cable (A) or at control lateral site (C). The timing of laser cut is indicated by the purple line.

(legend continued on next page)

development of apoptosensors (Baena-Lopez et al., 2018; Schott et al., 2017; Zhang et al., 2013), and (3) one can compare the same cell before and during apoptosis to identify differences between no-force and force generating cells.

Apico-basal force generation occurs at early stages of apoptosis. To date, characterization of cellular dynamics during cell elimination has essentially focused on cell extrusion from living tissues or cell monolayers in culture (Andrade and Rosenblatt, 2011; Eisenhoffer et al., 2012; Gu et al., 2011; Kuipers et al., 2014; Levayer et al., 2016; Marinari et al., 2012). Our combination of loss-of-function, time-lapse imaging and laser ablation experiments now shed light on early steps of apoptosis. Early apoptotic events include basal nucleus relocation and subsequent anchoring. The apico-basal force is next generated by cells committed to die through the formation of a continuum between their apical and basal surfaces. This continuum involves an apical contractile network composed of an actomyosin cable-like structure and a basal anchor composed of the nucleus, a non or poorly contractile F-actin cytoskeleton network and basal adhesions (Figure 7C).

Although commonly viewed as ''passive'', apoptotic cells are able to communicate with and modify their environment, both in physiological and pathological contexts (Ambrosini et al., 2017; Huang et al., 2011; Pérez-Garijo and Steller, 2015). Such communication, either chemical or physical, can be set up at stages where cells are committed to die, yet not destroyed. Understanding early steps of apoptosis is therefore of prime importance, but is a complex task since, during those steps, cells committed to die are barely distinguishable from their living neighbors. The development of apoptosensors adapted to live imaging allowed us to characterize the cellular machinery responsible for the orthogonal force produced by apoptotic cells *in vivo*, during a morphogenetic process. Our characterization of the apoptotic force machinery will provide a framework to better understand apico-basal forces in other morphogenetic processes, both in *Drosophila* and other model organisms.

Nucleus Relocation, an Unsuspected Early Step of Epithelial Cell Death

Chromatin remodeling, from its condensation to its fragmentation, has been extensively studied during apoptosis. Nucleus positioning, on the other hand, has been overlooked in dying cells. Although we have not characterized precisely the onset of nucleus basal relocation, it turned out to be one of the very first apoptotic events (with apical constriction) in a *Drosophila* epithelial sheet. Importantly, apoptotic nucleus relocation is unlikely to be restricted to morphogenetic apoptosis. Indeed, in the fly wing disc, upon death induction via irradiation, apoptotic nuclei are detected basally (Baena-Lopez et al., 2018). Hence, at least in fly imaginal tissues, early nuclear relocation is not specifically associated with morphogenetic apoptosis and could be a general prerequisite for cell destruction.

With this in mind, understanding the mechanistic basis of apoptotic nucleus motion is important. Our observations point toward an apoptotic LINC complex composed of Klarsicht and Klaroid. Klarsicht is known to associate with microtubules, although indirectly (Tapley and Starr, 2013). In both *Caenorhabditis elegans* and mouse, nesprins (Unc-83 and Syne-2 respectively) can associate with both dynein and kinesin complexes (Fridolfsson et al., 2010; Meyerzon et al., 2009; Zhang et al., 2009). A similar situation is likely at work in *Drosophila*. While pioneer works suggested a link between Klarsicht and the Dynein/Dynactin complex (Fischer et al., 2004), Klarsicht has been shown more recently to participate in a complex with and be localized by the plus-end motor kinesin-1(Gaspar et al., 2014). Epithelial cells are usually polarized with microtubules minus-end and plus-ends oriented toward their apical and basal pole respectively (Toya and Takeichi, 2016), suggesting that Klarsicht, associated with kinesin-1, could promote nucleus motion along lateral microtubule tracks in apoptotic cells. It will be of interest to determine the polarity of the non-centrosomal microtubule network in the leg disc and test whether dynein and kinesin can bind to klarsicht at the same time or whether their binding is mutually exclusive in dying cells. An appealing hypothesis would be that caspase activity could modulate binding properties or activity of klarsicht-associated motor proteins, leading to the basal relocation of apoptotic nucleus. Investigation of apoptotic nucleus motion could, at term, shed new light on the regulation of the LINC complex and on its association with the cytoskeleton.

(B and D) Stills extracted from movies showing sagittal views of the epithelium before (red) and after (green) laser cut at the level of the apico-basal myosin cable (B) or at a control lateral site (D). The myosin cable and the localization of the cut are shown by red and purple brackets respectively. Apico-basal recoil is observed in 13 out of 14 cases following cable cut (B) and 3 out of 17 following control lateral cut (D).

(E) Curves of the average apico-basal recoil +/− SEM over time (cable cut n = 14; control cut n = 17).

(F) Box plot representation of the apical surface release following apico-basal ablation in the indicated contexts. It corresponds to the variation in the angle made by the apical surface 25 s after ablation, compared to 1 s prior ablation (cable cut n = 15; control cut n = 17). Wilcoxon test: p value < 0.0002 (***).

(G and H) Stills extracted from a movie showing apoptotic nucleus behavior upon laser ablation of apico-basal myosin cable. (G) The DBS-S apoptosensor (cyan on the left panel) labels the nucleus in dying cells (arrow) and is enriched in the apical membrane in nonapoptotic cells. Myosin is shown in red and the apico-basal cable is highlighted by the white bracket. (H) Merge of DBS-S signal before (red) and after (green) myosin cable cut and associated schematization. Site of ablation is indicated by purple brackets. Individual images are shown in black and white on the left. The apoptotic nucleus moves basally after ablation in 9 cases out of 16, leading to an average basal displacement of 0.6 +/− 0.14 μm.

(I) Stills extracted from a movie showing nuclear envelope dynamics (red) of a dying cell (green) before (left) and during (right) the apoptotic nucleus upward movement associated with the force-generation stage. A close-up view of the apoptotic nuclear envelope is shown in black and schematized. Note that the apoptotic nucleus is apically deformed during the force-generation stage (arrowheads, n = 12/12). Asterisks identify the center of the nucleus, highlighting the upward nuclear movement (black arrow).

(J) Normalized index of nuclear envelope apical deformation before and during the force-generation stage represented as box plots (see STAR Methods; apoptotic nuclei, n = 12; nonapoptotic nuclei, n = 35). AU: arbitrary unit. Wilcoxon statistical test was used for the comparison of nuclei before and during force transmission; apoptotic nuclei (p value < 0.0005 for ***) and nonapoptotic nuclei (n.s., nonsignificant). Mann Whitney was used for comparison of apoptotic nuclei versus nonapoptotic nuclei before force transmission (n.s.).

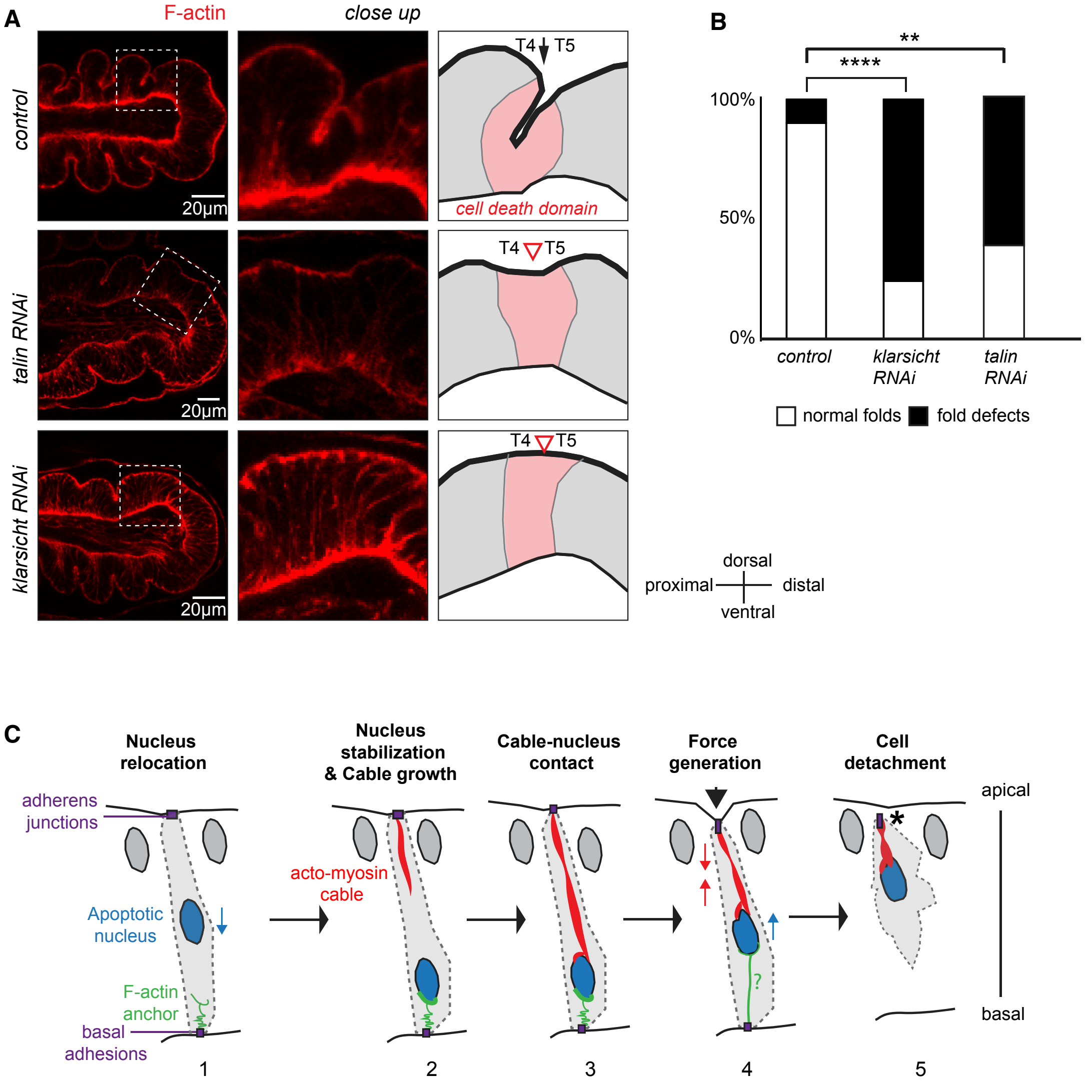

Figure 7. Perturbation of Apico-basal Force-Generation Affects Fold Formation

(A) General sagittal views of the distal leg (left), close up views on the T4/5 segments, and corresponding schemes of a control (top), *talin* RNAi (middle) and *klarsicht* RNAi (bottom) leg discs at 3–4 h after puparium formation. While deep folds form in the control (black arrow), shallow of absent folds are observed in knock-down contexts (open arrowheads).

(B) Histogram representation of the quantification of fold defects observed in control (n = 20), *klarsicht* RNAi (n = 37), and *talin* RNAi (n = 13) contexts. Fisher's statistical test: p values: <0.0001 for **** and <0.0036 for **.

(C) Model of the cellular organization responsible of apico-basal force generation in epithelial apoptotic cells: The apoptotic nucleus is first relocalized basally (1). It becomes anchored basally by an actin structure linked to basal adhesion (2). At the same stage, the Myosin II apico-basal cable starts its progression from the apical surface. Next, the myosin cable enters in contact with the nucleus, running along its apical surface (3). At this stage, basal adhesion, basal F-actin and the nucleus form a basal anchor to the cable. Subsequently, the Myosin II cable contracts deforming the apical surface of the epithelium and the apical side of the nucleus, thus transmitting force to the neighbors (4). Although the basal anchor is required at this stage, its organization is only speculative (question mark). Eventually, the force ends when the cell detaches from its neighbors (asterisk, 5).

The Nucleus, from an Organelle Subjected to Forces to a Key Component of the Force-Generation Machinery

An increasing body of work demonstrates that mechanical forces can affect many cellular behaviors. For example, differentiation of stem cells can be directed by mechanical properties of their substrate (or by activation of the Rho pathway) (Engler et al., 2006; McBeath et al., 2004; Yim and Sheetz, 2012). Differentiation of specialized structures such as cardiac valves are induced by blood flow (Boselli et al., 2015; Hove et al., 2003). Mesoderm stiffening can trigger the onset of collective migration of neural crest cells during *Xenopus* development (Barriga et al., 2018). During adulthood, compression forces caused by tumor growth can cause abnormal proliferation of adjacent, wild-type tissues, hence propagating the tumor (Fernández-Sánchez et al., 2015). Such responses necessitate that at some point, cells modify their gene expression program. Thus, mechanisms are set up to transfer information about cytoplasmic or external forces into the nucleus. For instance, compressive forces applied

upon fibroblast nuclei deform the nuclear envelope, modifying the shape of nucleopores, which allows entry of key molecules such as the transcription factor YAP into the nucleus (Elosegui-Artola et al., 2017). Alternatively, cells might use the LINC complex to transfer forces from the cytoskeleton to the nuclear envelope. Applying forces to the LINC (in isolated nuclei) leads to nucleus stiffening (Guilluy et al., 2014) while using a Nesprin-tension sensor shows that mechanical forces can indeed be transmitted across a nuclear membrane protein (Arsenovic et al., 2016). Moreover, compressive forces, for example forces generated when forcing cells to cross a small pore, can also change nucleus shape and transiently alter the integrity of its envelope, highlighting the importance of specialized repair mechanisms (Denais et al., 2016; Raab et al., 2016).

Since the primary function of the nucleus is to protect the genome and participate in the regulation of the genetic information, most of the studies addressing the relationship between the nucleus and cellular forces focused so far on how forces can trigger changes within the nucleus. Strikingly, our results demonstrate that, not only does the nucleus respond to forces, it also plays a key role in force generation at the cell scale during a morphogenetic process. The double connection with distinct cytoskeletal structures (one contractile and one resisting) switches the nucleus from being a moving organelle (Gundersen and Worman, 2013; Huelsmann et al., 2013; Lee and Norden, 2013; Zhao et al., 2012) to an essential constituent of the cellular machinery responsible for force generation. To our knowledge, this illustrates for the first time an additional, nongenomic function of the nucleus during animal development.

Pending questions are now whether the nucleus does participate in the cellular machinery responsible of apico-basal forces in other cell types, and to which extend does it participate in additional types of cellular forces. In this regard, it has been recently proposed that the nucleus can transmit forces from the rear to the front of isolated migrating cells in culture (Alam et al., 2015), suggesting the potential conservation of the nucleus as a key element in the force-generation machinery in different contexts.

STAR★METHODS

Detailed methods are provided in the online version of this paper and include the following:

- KEY RESOURCES TABLE
- CONTACT FOR REAGENT AND RESOURCE SHARING
- EXPERIMENTAL MODEL AND SUBJECT DETAILS
  - Breeding Conditions for Model Animals
  - Fly Stocks
- METHOD DETAILS
  - Immunostainings
  - Tissue Culture and Live Microscopy
  - Drug Treatment
  - Post-acquisition Isolation of Apoptotic Nuclei
  - Nuclei Positioning and Deformation
  - Laser Dissection
  - Analysis of Force Generation and Fold Morphogenesis
- QUANTIFICATION AND STATISTICAL ANALYSIS
  - Nuclei Positioning
  - Nuclei Stability
  - Break Duration during Baso-apical Nucleus Movement
  - Measures of Recoil after Laser Ablation
  - Quantification of Apical Nucleus Deformation

SUPPLEMENTAL INFORMATION

Supplemental Information can be found online at https://doi.org/10.1016/j.devcel.2019.05.027.

ACKNOWLEDGMENTS

The authors express their thanks to Mélanie Gettings, Anne Pelissier-Monier, and Alice Davy for helpful discussion, to Alberto Baena-Lopez for sharing reagents, and to the Imaging (LITC) and *Drosophila* facilities of the CBI. M.S.'s lab is supported by grants from the European Research Council (ERC) under the European Union Horizon 2020 research and innovation program (grant number EPAF: 648001), the Fondation Arc pour la Recherche sur le Cancer (CA 09-12-2014), and the Institut National de la Santé et de la Recherche Médicale (Inserm, Plan cancer 2014–2019). A.A. has been supported by the French Ministry of Research and A.A. and M.R. by la Ligue Nationale contre le Cancer during their PhD.

AUTHOR CONTRIBUTIONS

A.A. and M.R. performed most experiments and data analysis. M.R., B.M., and M.S. performed ablation experiments. B.M. and M.S. supervised the project. Funding acquisition: M.S.

DECLARATION OF INTERESTS

The authors declare no competing interests.

Received: November 5, 2018
Revised: March 11, 2019
Accepted: May 10, 2019
Published: June 13, 2019

REFERENCES

Alam, S.G., Lovett, D., Kim, D.I., Roux, K.J., Dickinson, R.B., and Lele, T.P. (2015). The nucleus is an intracellular propagator of tensile forces in NIH 3T3 fibroblasts. J. Cell Sci. *128*, 1901–1911.

Ambrosini, A., Gracia, M., Proag, A., Rayer, M., Monier, B., and Suzanne, M. (2017). Apoptotic forces in tissue morphogenesis. Mech. Dev. *144*, 33–42.

Andrade, D., and Rosenblatt, J. (2011). Apoptotic regulation of epithelial cellular extrusion. Apoptosis *16*, 491–501.

Arsenovic, P.T., Ramachandran, I., Bathula, K., Zhu, R., Narang, J.D., Noll, N.A., Lemmon, C.A., Gundersen, G.G., and Conway, D.E. (2016). Nesprin-2G, a component of the nuclear LINC complex, is subject to myosin-dependent tension. Biophys. J. *110*, 34–43.

Baena-Lopez, L.A., Arthurton, L., Bischoff, M., Vincent, J.P., Alexandre, C., and McGregor, R. (2018). Novel initiator caspase reporters uncover previously unknown features of caspase-activating cells. Development *145*.

Barriga, E.H., Franze, K., Charras, G., and Mayor, R. (2018). Tissue stiffening coordinates morphogenesis by triggering collective cell migration in vivo. Nature *554*, 523–527.

Boselli, F., Freund, J.B., and Vermot, J. (2015). Blood flow mechanics in cardiovascular development. Cell. Mol. Life Sci. *72*, 2545–2559.

Brodland, G.W., Conte, V., Cranston, P.G., Veldhuis, J., Narasimhan, S., Hutson, M.S., Jacinto, A., Ulrich, F., Baum, B., and Miodownik, M. (2010). Video force microscopy reveals the mechanics of ventral furrow invagination in Drosophila. Proc. Natl. Acad. Sci. USA *107*, 22111–22116.

Chanet, S., and Martin, A.C. (2014). Mechanical force sensing in tissues. Prog. Mol. Biol. Transl. Sci. *126*, 317–352.

Chung, S., Kim, S., and Andrew, D.J. (2017). Uncoupling apical constriction from tissue invagination. ELife *6*.

Colas, J.F., and Schoenwolf, G.C. (2001). Towards a cellular and molecular understanding of neurulation. Dev. Dyn. *221*, 117–145.

Coravos, J.S., and Martin, A.C. (2016). Apical sarcomere-like actomyosin contracts nonmuscle drosophila epithelial cells. Dev. Cell *39*, 346–358.

Davidson, L.A. (2012). No strings attached: new insights into epithelial morphogenesis. BMC Biol. *10*, 105.

Denais, C.M., Gilbert, R.M., Isermann, P., McGregor, A.L., te Lindert, M., Weigelin, B., Davidson, P.M., Friedl, P., Wolf, K., and Lammerding, J. (2016). Nuclear envelope rupture and repair during cancer cell migration. Science *352*, 353–358.

Eisenhoffer, G.T., Loftus, P.D., Yoshigi, M., Otsuna, H., Chien, C.B., Morcos, P.A., and Rosenblatt, J. (2012). Crowding induces live cell extrusion to maintain homeostatic cell numbers in epithelia. Nature *484*, 546–549.

Elosegui-Artola, A., Andreu, I., Beedle, A.E.M., Lezamiz, A., Uroz, M., Kosmalska, A.J., Oria, R., Kechagia, J.Z., Rico-Lastres, P., Le Roux, A.L., et al. (2017). Force triggers YAP nuclear entry by regulating transport across nuclear pores. Cellule *171*, 1397–1410.

Engler, A.J., Sen, S., Sweeney, H.L., and Discher, D.E. (2006). Matrix elasticity directs stem cell lineage specification. Cell *126*, 677–689.

Fernández-Sánchez, M.E., Barbier, S., Whitehead, J., Béalle, G., Michel, A., Latorre-Ossa, H., Rey, C., Fouassier, L., Claperon, A., Brullé, L., et al. (2015). Mechanical induction of the tumorigenic β-catenin pathway by tumour growth pressure. Nature *523*, 92–95.

Fischer, J.A., Acosta, S., Kenny, A., Cater, C., Robinson, C., and Hook, J. (2004). Drosophila klarsicht has distinct subcellular localization domains for nuclear envelope and microtubule localization in the eye. Genetics *168*, 1385–1393.

Fridolfsson, H.N., Ly, N., Meyerzon, M., and Starr, D.A. (2010). UNC-83 coordinates kinesin-1 and dynein activities at the nuclear envelope during nuclear migration. Dev. Biol. *338*, 237–250.

Gaspar, I., Yu, Y.V., Cotton, S.L., Kim, D.H., Ephrussi, A., and Welte, M.A. (2014). Klar ensures thermal robustness of oskar localization by restraining RNP motility. J. Cell Biol. *206*, 199–215.

Gu, Y., Forostyan, T., Sabbadini, R., and Rosenblatt, J. (2011). Epithelial cell extrusion requires the sphingosine-1-phosphate receptor 2 pathway. J. Cell Biol. *193*, 667–676.

Guilluy, C., Osborne, L.D., Van Landeghem, L., Sharek, L., Superfine, R., Garcia-Mata, R., and Burridge, K. (2014). Isolated nuclei adapt to force and reveal a mechanotransduction pathway in the nucleus. Nat. Cell Biol. *16*, 376–381.

Gundersen, G.G., and Worman, H.J. (2013). Nuclear positioning. Cell *152*, 1376–1389.

He, B., Doubrovinski, K., Polyakov, O., and Wieschaus, E. (2014). Apical constriction drives tissue-scale hydrodynamic flow to mediate cell elongation. Nature *508*, 392–396.

Heisenberg, C.P., and Bellaïche, Y. (2013). Forces in tissue morphogenesis and patterning. Cell *153*, 948–962.

Hildebrand, J.D. (2005). Shroom regulates epithelial cell shape via the apical positioning of an actomyosin network. J. Cell Sci. *118*, 5191–5203.

Hove, J.R., Köster, R.W., Forouhar, A.S., Acevedo-Bolton, G., Fraser, S.E., and Gharib, M. (2003). Intracardiac fluid forces are an essential epigenetic factor for embryonic cardiogenesis. Nature *421*, 172–177.

Huang, Q., Li, F., Liu, X., Li, W., Shi, W., Liu, F.F., O'Sullivan, B., He, Z., Peng, Y., Tan, A.C., et al. (2011). Caspase 3-mediated stimulation of tumor cell repopulation during cancer radiotherapy. Nat. Med. *17*, 860–866.

Huelsmann, S., Ylänne, J., and Brown, N.H. (2013). Filopodia-like actin cables position nuclei in association with perinuclear actin in Drosophila nurse cells. Dev. Cell *26*, 604–615.

Keller, R., Davidson, L.A., and Shook, D.R. (2003). How we are shaped: the biomechanics of gastrulation. Differentiation *71*, 171–205.

Kiehart, D.P. (2015). Epithelial morphogenesis: apoptotic forces drive cell shape changes. Dev. Cell *32*, 532–533.

Klapholz, B., and Brown, N.H. (2017). Talin - the master of integrin adhesions. J. Cell Sci. *130*, 2435–2446.

Kondo, T., and Hayashi, S. (2015). Mechanisms of cell height changes that mediate epithelial invagination. Dev. Growth Differ. *57*, 313–323.

Kuipers, D., Mehonic, A., Kajita, M., Peter, L., Fujita, Y., Duke, T., Charras, G., and Gale, J.E. (2014). Epithelial repair is a two-stage process driven first by dying cells and then by their neighbours. J. Cell Sci. *127*, 1229–1241.

Lecuit, T., Lenne, P.F., and Munro, E. (2011). Force generation, transmission, and integration during cell and tissue morphogenesis. Annu. Rev. Cell Dev. Biol. *27*, 157–184.

Lee, H.O., and Norden, C. (2013). Mechanisms controlling arrangements and movements of nuclei in pseudostratified epithelia. Trends Cell Biol. *23*, 141–150.

Lee, Y.L., and Burke, B. (2018). LINC complexes and nuclear positioning. Semin. Cell Dev. Biol. *82*, 67–76.

Levayer, R., Dupont, C., and Moreno, E. (2016). Tissue crowding induces caspase-dependent competition for space. Curr. Biol. *26*, 670–677.

Liang, X., Michael, M., and Gomez, G.A. (2016). Measurement of mechanical tension at cell-cell junctions using two-photon laser ablation. Bio Protoc. *6*.

Lubkov, V., and Bar-Sagi, D. (2014). E-cadherin-mediated cell coupling is required for apoptotic cell extrusion. Curr. Biol. *24*, 868–874.

Manjón, C., Sánchez-Herrero, E., and Suzanne, M. (2007). Sharp boundaries of Dpp signalling trigger local cell death required for Drosophila leg morphogenesis. Nat. Cell Biol. *9*, 57–63.

Marinari, E., Mehonic, A., Curran, S., Gale, J., Duke, T., and Baum, B. (2012). Live-cell delamination counterbalances epithelial growth to limit tissue overcrowding. Nature *484*, 542–545.

Martin, A.C., Gelbart, M., Fernandez-Gonzalez, R., Kaschube, M., and Wieschaus, E.F. (2010). Integration of contractile forces during tissue invagination. J. Cell Biol. *188*, 735–749.

Martin, A.C., and Goldstein, B. (2014). Apical constriction: themes and variations on a cellular mechanism driving morphogenesis. Development *141*, 1987–1998.

Martin, A.C., Kaschube, M., and Wieschaus, E.F. (2009). Pulsed contractions of an actin-myosin network drive apical constriction. Nature *457*, 495–499.

Mason, F.M., Tworoger, M., and Martin, A.C. (2013). Apical domain polarization localizes actin-myosin activity to drive ratchet-like apical constriction. Nat. Cell Biol. *15*, 926–936.

McBeath, R., Pirone, D.M., Nelson, C.M., Bhadriraju, K., and Chen, C.S. (2004). Cell shape, cytoskeletal tension, and RhoA regulate stem cell lineage commitment. Dev. Cell *6*, 483–495.

Meyerzon, M., Fridolfsson, H.N., Ly, N., McNally, F.J., and Starr, D.A. (2009). UNC-83 is a nuclear-specific cargo adaptor for kinesin-1-mediated nuclear migration. Development *136*, 2725–2733.

Monier, B., Gettings, M., Gay, G., Mangeat, T., Schott, S., Guarner, A., and Suzanne, M. (2015). Apico-basal forces exerted by apoptotic cells drive epithelium folding. Nature *518*, 245–248.

Owaribe, K., Kodama, R., and Eguchi, G. (1981). Demonstration of contractility of circumferential actin bundles and its morphogenetic significance in pigmented epithelium in vitro and in vivo. J. Cell Biol. *90*, 507–514.

Pearl, E.J., Li, J., and Green, J.B. (2017). Cellular systems for epithelial invagination. Philos. Trans. R. Soc. Lond. B Biol. Sci. *372*.

Pérez-Garijo, A., and Steller, H. (2015). Spreading the word: non-autonomous effects of apoptosis during development, regeneration and disease. Development *142*, 3253–3262.

Raab, M., Gentili, M., de Belly, H., Thiam, H.R., Vargas, P., Jimenez, A.J., Lautenschlaeger, F., Voituriez, R., Lennon-Duménil, A.M., Manel, N., et al. (2016). ESCRT III repairs nuclear envelope ruptures during cell migration to limit DNA damage and cell death. Science *352*, 359–362.

Roh-Johnson, M., Shemer, G., Higgins, C.D., McClellan, J.H., Werts, A.D., Tulu, U.S., Gao, L., Betzig, E., Kiehart, D.P., and Goldstein, B. (2012). Triggering a cell shape change by exploiting preexisting actomyosin contractions. Science *335*, 1232–1235.

Rosenblatt, J., Raff, M.C., and Cramer, L.P. (2001). An epithelial cell destined for apoptosis signals its neighbors to extrude it by an actin- and myosin-dependent mechanism. Curr. Biol. *11*, 1847–1857.

Schott, S., Ambrosini, A., Barbaste, A., Benassayag, C., Gracia, M., Proag, A., Rayer, M., Monier, B., and Suzanne, M. (2017). A fluorescent toolkit for spatio-temporal tracking of apoptotic cells in living Drosophila tissues. Development *144*, 3840–3846.

Sherrard, K., Robin, F., Lemaire, P., and Munro, E. (2010). Sequential activation of apical and basolateral contractility drives ascidian endoderm invagination. Curr. Biol. *20*, 1499–1510.

Tapley, E.C., and Starr, D.A. (2013). Connecting the nucleus to the cytoskeleton by SUN-KASH bridges across the nuclear envelope. Curr. Opin. Cell Biol. *25*, 57–62.

Toya, M., and Takeichi, M. (2016). Organization of non-centrosomal microtubules in epithelial cells. Cell Struct. Funct. *41*, 127–135.

Umetsu, D., and Kuranaga, E. (2017). Planar polarized contractile actomyosin networks in dynamic tissue morphogenesis. Curr. Opin. Genet. Dev. *45*, 90–96.

Yang, Q., Roiz, D., Mereu, L., Daube, M., and Hajnal, A. (2017). The invading anchor cell induces lateral membrane constriction during vulval lumen morphogenesis in C. elegans. Dev. Cell *42*, 271–285.

Yim, E.K., and Sheetz, M.P. (2012). Force-dependent cell signaling in stem cell differentiation. Stem Cell Res. Ther. *3*, 41.

Zhang, J., Wang, X., Cui, W., Wang, W., Zhang, H., Liu, L., Zhang, Z., Li, Z., Ying, G., Zhang, N., et al. (2013). Visualization of caspase-3-like activity in cells using a genetically encoded fluorescent biosensor activated by protein cleavage. Nat. Commun. *4*, 2157.

Zhang, X., Lei, K., Yuan, X., Wu, X., Zhuang, Y., Xu, T., Xu, R., and Han, M. (2009). Sun1/2 and Syne/Nesprin-1/2 complexes connect centrosome to the nucleus during neurogenesis and neuronal migration in mice. Neuron *64*, 173–187.

Zhao, T., Graham, O.S., Raposo, A., and St Johnston, D. (2012). Growing microtubules push the oocyte nucleus to polarize the Drosophila dorsal-ventral axis. Science *336*, 999–1003.

STAR★METHODS

KEY RESOURCES TABLE

REAGENT or RESOURCE	SOURCE	IDENTIFIER
Antibodies		
Mouse anti-klarsicht-C (1:50)	Developmental Studies Hybridoma Bank (DSHB)	9C10; RRID: AB_10584797
Mouse anti-Lamin Dm0 (1:50)	DSHB	ADL195; RRID: AB_528333
Rat anti-E-Cadherin (1:50)	DSHB	DCAD2; RRID: AB_528120
Rabbit Anti-cleaved Dcp1 (1:200)	Cell Signaling Technologies	Cat#9578; RRID: AB_2721060
Chemicals, Peptides, and Recombinant Proteins		
Vectashield with DAPI	Vector Laboratories	Cat#H-1200
Phalloidin-Rhodamine	Fisher Scientific	Cat#10063052
FM4-64	Fisher Scientific	Cat#10717864
Cytochalasin D	Sigma-Aldrich	Cat#C2618
DMSO	Sigma-Aldrich	Cat#D8418
Hoechst 33342	Sigma-Aldrich	Cat#B2883
20-hydroxyecdysone	Sigma-Aldrich	Cat# H5142
Agarose type VII	Sigma-Aldrich	Cat#A4018
Experimental Models: Organisms/Strains		
D. melanogaster: Myosin-GFP: *w, sqh{TI}-eGFP [29B]*	This study	N/A
D. melanogaster: Myosin-TagRFPt: *w, sqh{TI}-TagRFPt [3B]*	This study	N/A
D. melanogaster: w, lamin{TI}-TagRFPt	This study	N/A
D. melanogaster: y, w; rhea[MI00296]-GFP/TM6B	BDSC	RRID: BDSC_39649
D. melanogaster: y,w; rhea[MI00296]-mCherry/TM6B	BDSC	RRID: BDSC_39648
D. melanogaster: w; UAS::alpha-Catenin-TagRFP	Gift from K. Sugimura	FBal0279996 Kyoto University, iCeMS
D. melanogaster: y, w, cv, sqh[AX3]; sqh::sqh-GFP	BDSC	RRID: BDSC_57144
D. melanogaster: y, w; klaroid[CB04483]-GFP	BDSC	RRID: BDSC_51525
D. melanogaster: y, w; Map205[CC00109]-GFP	BDSC	RRID: BDSC_51533
D. melanogaster: w; arm::arm-GFP	BDSC	RRID: BDSC_8556
D. melanogaster: y, w; UAS::lamin-GFP	BDSC	RRID: BDSC_7378
D. melanogaster: y, w, HS::Flp; act5C-Flp-y+-FRT::Gal4, UAS::GFP	Monier et al., 2015	Centre de Biologie Intégrative (CBI), LBCMCP
D. melanogaster: w; ; UAS::His2Av-mKO	BDSC	RRID: BDSC_53731
D. melanogaster: w; ; UAS::lifeact-TagRFPt	BDSC	RRID: BDSC_58714
D. melanogaster: w; ; UAS::mcd8-mCherry	BDSC	RRID: BDSC_27392
D. melanogaster: cytoplasmic GFP apoptosensor: w; UAS::GC3Ai [G16]	Schott et al., 2017	Centre de Biologie Intégrative (CBI), LBCMCP
D. melanogaster: cytoplasmic GFP apoptosensor: w; ; UAS::GC3Ai [G75]	Schott et al., 2017	Centre de Biologie Intégrative (CBI), LBCMCP
D. melanogaster: w; UAS::Utrophin-GFP	Gift from T. Lecuit	FBtp0073094
D. melanogaster: w; Msp300[CPTI003472]-Venus	DGRC Kyoto	FBst0325397
D. melanogaster: Dll::Gal4[MD23]/CyO	BDSC	RRID: BDSC_3038
D. melanogaster: y, w; ap::Gal4[md544]/CyO	BDSC	RRID: BDSC_3041
D. melanogaster: y, sc, v; ; UAS::RNAi-klarsicht[HMS01612]	BDSC	RRID: BDSC_36721
D. melanogaster: y, v; ; UAS::RNAi-klarsicht[JF02944], e	BDSC	RRID: BDSC_28313
D. melanogaster: y, sc, v; ; UAS::RNAi-rhea[HMS00856]	BDSC	RRID: BDSC_33913
D. melanogaster: w; ; UAS::RNAi-rhea[40339]	VDRC	V40339; FBst0463536
D. melanogaster: y, sc, v; UAS::RNAi-lamin[HMC04816]	BDSC	RRID: BDSC_57501

(*Continued on next page*)

Continued		
REAGENT or RESOURCE	SOURCE	IDENTIFIER
D. melanogaster: w; koi[80]	BDSC	RRID: BDSC_25105
D. melanogaster: nuclear GFP apoptosensor: *w; tub::DBS-S*	Gift from A. Baena-Lopez; Baena-Lopez et al., 2018	Dunn School, University of Oxford
Software and Algorithms		
Prism 8	GraphPad	RRID: SCR_002798
Photoshop CS5	Adobe	RRID: SCR_014199
Imaris 8.4.1	Bitplane	RRID: SCR_007370
Fiji	https://fiji.sc/	RRID: SCR_002285
Other		
120 μm deep Secure-Seal™	Sigma-Aldrich	Cat#GBL654008
Shields and Sang M3	Sigma-Aldrich	Cat#S3652
Schneider's insect medium	Sigma-Aldrich	Cat#S0146
Halocarbon oil	Sigma-Aldrich	Cat#H8773
Microscopes		
Confocal LSM880	Zeiss	54477
CellObserver Spinning Disc	Zeiss	57174
Objective 40X 1.4 Oil Plan APO	Zeiss	4207 62 - 9900
Objective 40X 1.2 Water C APO	Zeiss	421767-9971-790
Objective 63X 1.4 Oil APO	Zeiss	420782 - 9900
Objective 63X 1.2 Water C APO Dic	Zeiss	421787 - 9971 - 790
Pulsed Laser 532nm	Rapp-opto	DPSL 532/42/CLS UGA-42 Firefly

CONTACT FOR REAGENT AND RESOURCE SHARING

Further information and requests for resources and reagents should be directed to and will be fulfilled by the Lead Contact, Magali Suzanne (magali.suzanne@univ-tlse3.fr).

EXPERIMENTAL MODEL AND SUBJECT DETAILS

Breeding Conditions for Model Animals

The animal model used here is Drosophila melanogaster, in a context of *in vivo/ex vivo* experiments. In order to respect ethic principles, animals were anesthetized with CO2 (adults) before any manipulation. To avoid any release of flies outside the laboratory, dead flies were frozen before throwing them. Stocks of living flies were conserved in incubators, either at 18 or 25 degrees to maintain the flies in optimal condition. Genotypes and developmental stages are indicated below. Experiments were performed in both males and females indifferently. Loss of function experiments using RNAi were carried out at 30 degrees.

Fly Stocks

Lamin-TagRFPt, sqh-eGFP[29B] and sqh-TagRFPt[3B] (this work) are knock-in designed and generated by homologous recombination by InDroso functional genomics (Rennes, France). The respective tags were inserted in C-terminal just before the stop codon for sqh and in N-terminal just before the ATG for lamin, and the resulting flies were validated by sequencing.

Additional fluorescent lines obtained from Bloomington Drosophila Stock Center (BDSC) are: rhea[MI00296]-GFP, rhea[MI00296]-mCherry, sqh[AX3]; sqh::sqh-GFP, klaroid[CB04483]-GFP, Map205[CC00109]-GFP, arm::arm-GFP, UAS::lamin-GFP, UAS::His2Av-mKO, UAS::lifeactTagRFPt, UAS::mcd8-mCherry. The GFP apoptosensor UAS::GC3Ai was described previously (Schott et al., 2017). The tub::DBS-S apoptosensor, UAS::α-catenin-TagRFP and UAS::Utrophin-GFP are gifts from A. Baena-Lopez, K. Sugimura and T. Lecuit respectively. Msp300[CPTI003472]-Venus was obtained from DGRC (Kyoto, Japan). Additional stocks are Dll::Gal4 [MD23], ap::Gal4[md544], act5C-Flp-y+-FRT::Gal4, UAS::GFP and HS::Flp.

RNA interference was realized using UAS::RNAi-klarsicht[HMS01612] and UAS::RNAi-klarsicht[JF02944] (both lines were obtained from BDSC and gave similar results). UAS::RNAi-rhea[HMS00856] and UAS::RNAi-rhea[v40339] (obtained from BDSC and Vienna Drosophila Resource Center, both lines gave similar results) were used to knock-down Talin. UAS::RNAi-lamin [HMC04816] and koi[80] mutant line were obtained from BDSC.

METHOD DETAILS

Immunostainings

Leg discs from prepupae (from 0h to 4h after puparium formation depending on experiments) are dissected in PBS 1x. Tissue are fixed by paraformaldehyde 4% diluted in PBS 1x during 20 minutes. Then the samples are washed and saturated in PBS 1x, 0.3% triton x-100 and BSA 1% (BBT). Next, the samples are incubated overnight at 4°C with primary antibodies diluted in BBT. Samples are washed for 1h in BBT before a 2h incubation at room temperature with secondary antibodies diluted in BBT. Finally, samples are washed with PBS 1x, 0.3% Triton x-100 for 1h and mounted in Vectashields containing DAPI (Vector Laboratories). A 120-μm-deep spacer (Secure-Seal™ from Sigma-Aldrich) is placed in between the glass slide and the coverslip to preserve morphology of the tissues.

Primary antibodies from Developmental Studies Hybridoma Bank (DSHB) are klarsicht-C antibody (9C10-s, mouse, 1:50), Lamin Dm0 (ADL195-s, mouse, 1:50) and E-Cadherin antibody (DCAD2, rat, 1:50). Anti-cleaved Dcp-1 (#9578, rabbit, 1:200) was obtained from Cell Signaling Technologies. Secondary antibodies (Alexa488 and 647) are purchased from Interchim and diluted at 1:200 or 1:100 respectively. Phalloidine-Rhodamine (Fischer Scientific) used to stain F-actin is diluted at 1:200.

Tissue Culture and Live Microscopy

Leg discs are dissected at white pupal stage in Shields and Sang M3 or Schneider's insect medium (Sigma-Aldrich) supplemented with 15 % fetal calf serum and 0.5 % penicillin-streptomycin as well as 20-hydroxyecdysone at 2 μg/mL (Sigma-Aldrich, H5142). Leg discs are transferred on a glass slide in 13.5 μL of this medium confined in a 120 μm-deep double-sided adhesive spacer (Secure-SealTM from Sigma-Aldrich). A glass coverslip is then placed on top of the spacer. Halocarbon oil is added on the sides of the spacer to prevent dehydration. Dissection tools are cleaned with ethanol before dissection.

Nuclei are labelled *in vivo* either through expression of a UAS::His2Av-mKO construct or using Hoechst staining. For this purpose, dissected disc are incubated in the dark for 20 minutes with Hoechst 33342 (Sigma, B2883, 25mg/ml) diluted at (1:100) in the culture medium. Legs are then transferred in fresh medium without Hoechst and mounted between slide and coverslip as described above. To avoid Hoechst toxicity, fly tissues are imaged for no longer than 1h30. Membrane staining using FM4-64 was performed as described in Monier et al. (2015).

Live and fixed samples were imaged using either an inverted LSM880 Zeiss confocal mounted with 40x/1.2 or 63x/1.3 water objectives or 40x/1.4 or 63x/1.4 oil objectives and equipped with a piezo stage or using an inverted spinning disk confocal microscope (CSU-X1, Yokogawa, coupled to a Leica or a Zeiss microscope) equipped with 405 nm, 488 nm and 561 nm LEDs, a piezo stage and a Hamamatsu EMCCD camera controlled by the Metamorph or zen softwares. Images were processed with the Fiji and Imaris softwares.

Drug Treatment

Cytochalasin D is a chemical agent which causes F-actin depolymerisation. We set up conditions that reduces F-actin levels without altering tissue integrity. Following dissection, leg discs are incubated during 20 minutes with Cytochalasin D (Sigma; #C2618, 5μg/ml) diluted at 1:1000 in Shields and Sang medium. DMSO was used for control conditions. Tissues are next washed with fresh medium and mounted between a glass slide and a coverslip separated with a spacer. The spacer is partly filled with a thin layer (around 5μm) of low gelling temperature agarose (Sigma, Agarose type VII, #A4018), 0,25% final diluted in Shields and Sang medium. 6μl of medium are added on top of agarose and leg discs are transferred (with a drop of about 4μl of medium).

Post-acquisition Isolation of Apoptotic Nuclei

In order to follow in the same cell the dynamics of both the nucleus and the myosin cable, it is necessary to acquire a z-stack encompassing the dying cell. However, cell packing renders nuclei tracking in 3D stacks complex. To circumvent this difficulty, we applied post-acquisition image treatment on movies obtained with confocal LSM880 (Zeiss). To increase spatial resolution, we used a ROI on apoptotic cells stained with the GFP cytoplasmic apoptosensor (Schott et al., 2017), while nuclei are labelled with Hoechst and myosin using the knock-in line sqh-TagRFPt. Images are acquired every 20 to 30 seconds for at least 20 minutes. Next, we perform processing of the movies using the IMARIS software. We first create a 3D surface on the apoptotic cell staining. This is used to generate a mask which allows to specifically reveal the nucleus staining of the dying cell. This process is used in Figures 5A and 5B and illustrated in Figure S4.

Nuclei Positioning and Deformation

To analyze nuclei dynamics during basal stabilization phase (Figures 4D and 4G), we performed movies with high temporal resolution in short periods of time using spinning disk microscopes. Images are taken on one single plane every 2 seconds during 5 minutes on legs co-expressing UAS::Histone2Av-mKO and UAS::GC3Ai transgenes that respectively label nuclei and apoptotic cells (see quantification in statistics section). We next used the MTrackJ plug in of the ImageJ software to manually track apoptotic and non-apoptotic nuclei and measure their velocity (Figure 4D). Alternatively, we used Imaris to track nuclei by creating spots on Histone2Av-mko staining (on both apoptotic nuclei and non-apoptotic nuclei) (Figure 4G).

To extract the behavior of the nucleus in control and RNAi *talin* conditions at the force stage (Figures 5C and 5D), we used the IMARIS software. We generated a spot on the isolated apoptotic nucleus (see "Post-acquisition isolation of apoptotic nuclei" section) and created a track of nuclear movement. Nuclei dynamics were visualized using Imaris speed color-coded scale.

To study nuclear distortion during force generation, z-stack (~7μm) were acquired every 10 seconds during 30 minutes (time estimated to visualize upward nuclear movement) on legs expressing Lamin-tagRFPt and UAS::GC3Ai transgenes that respectively label nuclear envelope and apoptotic cells (Figures 6I and 6J) (see quantifaction and statistics section). We used a Zeiss spinning disc microscope.

Laser Dissection

Leg discs were dissected from white pupae (0-1 h APF), and mounted between a glass slide and a coverslip separated by a spacer to preserve leg morphology. Laser dissection experiments (Figures 6A–6H) were performed on a Zeiss LSM880 laser scanning microscope fitted with a pulsed DPSS laser (532 nm, pulse length 1.5 ns, repetition rate up to 1 kHz, 3.5 μJ/pulse) steered by a galvanometer-based laser scanning device (DPSS-532 and UGA-42, from Rapp OptoElectronic, Hamburg, Germany). The laser beam was focused through a water-immersion lens of high numerical aperture (Plan-Apochromat 63x from Zeiss). Experiments were performed using a numerical 2x zoom. Photo-disruption in the middle of the leg epithelium was produced in the focal plane by illuminating at 100 % laser power a 70 pixel line (around 10μm) for 5s. Images of Myosin-GFP or DBS-S were acquired every 500 ms to 1s using a 488 nm Argon laser and a GaAsP photomultiplier. In the case of DBS-S, the green apoptosensor was crossed to Myosin-TagRFPt to visualize the stage of the apoptotic cell and determine the region to be ablated. Due to photobleaching caused the 532 nm pulsed laser, Myosin-TagRFPt could not be analyzed after laser cut. Cells were selected for their apico-basal myosin cable spanning about the half of the epithelium and their localization in the presumptive T4/T5 fold region, and for the additional presence of nuclear GFP in the case of the DBS apoptosensor.

Analysis of Force Generation and Fold Morphogenesis

For analysis of apico-basal force generation (Figures 3H and 5G), we performed time-lapse movies of cells co-expressing the α-catenin-RFP and the apoptotsensor under expression of the apterous::Gal4 driver. We counted the percentage of cells able to deform the apical surface of the epithelium in control, klarsicht RNAi and *talin* RNAi.

For analysis of morphogenesis defects (Figure 7B), we fixed pupae of 3-4h APF. UAS::RNAi were driven by Distalless::Gal4. We stained legs with phalloidin to reveal the overall morphology and counted the number of legs with affected folds (shallow, twisted, absent). For both types of experiments, we performed the fisher statistic test to determine if differences between samples were significant.

QUANTIFICATION AND STATISTICAL ANALYSIS

Statistics were performed in Prism. N and p values are indicated in figure legends. Box plot were generated in Prism and represent the median, 10 and 90 percentile.

Nuclei Positioning

Related to Figures 2D, 3C, and 3E. The position of nuclei along the apico-basal axis was determined by calculating the ratio of the distance from nucleus center to basal surface divided by the overall cell height. Distances were measured using the Zen program (Zeiss). We used the Wilcoxon test for paired samples in Figure 2D and the Mann Whitney test for non-paired samples in Figures 3C and 3E.

Lamin Levels

Related to Figure S1B. Lamin levels were measured using Fiji by drawing a segmented line of the width of the nuclear envelope on single z planes. In each leg, the averaged intensity of non-apoptotic nuclei was used to normalize the intensity of each individual nucleus. We performed the Mann Whitney test because this is a non-parametric test with non paired samples.

Nuclei Stability

Related to Figures 4D and 4G. Using ImageJ or Imaris softwares, we measured the displacement of nuclei per minute. For comparison of apoptotic and non-apoptotic nuclei in DMSO (Figure 4D) or in control condition (Figure 4G), we calculated the mean velocity of apoptotic and non-apoptotic nuclei per leg and compared samples using paired Wilcoxon-test. For other comparisons, we performed the Mann Whitney test because this is a non-parametric test with non paired samples.

Break Duration during Baso-apical Nucleus Movement

Related to Figure 5E. The break in upward motion of apoptotic nuclei at the force-generation stage was determined on the tracks of nuclei movement generated by the Imaris software (see above). Beginning and end of the break phase were determined based on the change of nuclei velocity and direction. The difference of the break in nuclei upward motion was compared using Mann Whitney test.

Measures of Recoil after Laser Ablation

Related to Figures 6E, 6F, and 6H. Quantification of recoil after ablation of Myosin-GFP apico-basal cables or control ablation of lateral membranes was performed by following two intense myosin structures over time as described in (Liang et al., 2016). Kymographs were generated using Fiji. Quantification of basal nuclear movement was achieved on the DBS-S apoptosensor (Baena-Lopez et al., 2018). Images we registered using the Fiji stackreg plug in. The displacement of the apoptotic nucleus from the site of ablation was then measured at around 30 seconds after the laser cut.

Quantification of the recoil of the apical surface was achieved in Fiji by measuring the angle made by the apical surface (stained by Myosin-GFP) above the site of lateral ablation. We calculated the difference between the apical surface angle before (-1 sec) and after (+25 sec) laser cut.

Quantification of Apical Nucleus Deformation

Related to Figure 6J. The local transient apical deformation of the apoptotic nucleus was determined in Fiji. We first fitted the closest ellipse to the nucleus. Next, we draw a polygon that follows the outline of the apical region of the nucleus. The fit spline option was used to make the polygon smooth. The apical nucleus deformation corresponds to the difference of surface between the polygon and the ellipse which is normalized by the surface of the ellipse and expressed as a percentage. We compared apoptotic nucleus shape just prior and during baso-apical movement (which corresponds to the force stage); non-apoptotic nuclei were analyzed on the same images. Wilcoxon statistical test was used for the comparison of nuclei before and during force transmission; apoptotic nuclei (p-value <0.0005 for ***) and non-apoptotic nuclei (n.s.: non significant). Mann Whitney was used for comparison of apoptotic nulei vs non apoptotic nulei before force transmission (n.s.).

Developmental Cell, Volume *50*

Supplemental Information

Mechanical Function of the Nucleus in Force Generation during Epithelial Morphogenesis

Arnaud Ambrosini, Mégane Rayer, Bruno Monier, and Magali Suzanne

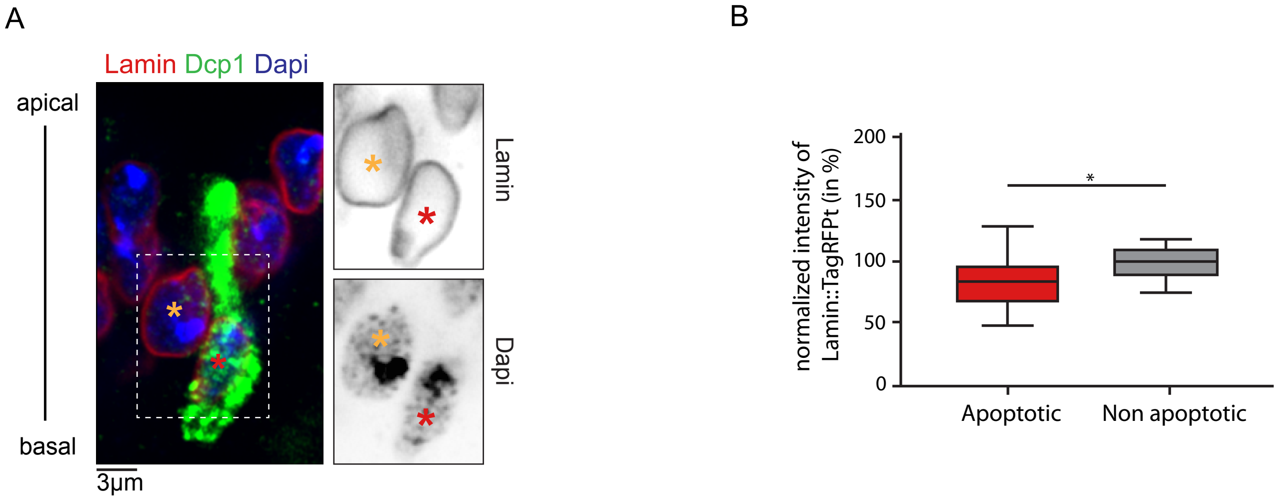

Figure S1. Nucleus morphology and integrity in early apoptotic cells
(A) Sagittal section of a fixed prepupal leg (left). Chromatin (DAPI) is in blue, nuclear envelope (Lamin) in red. An apoptotic cell is visualized by anti-Dcp1 staining (green). Close up views of Lamin (top) and DAPI (bottom) are shown on the right. Lamin distribution appears similar in apoptotic (red star) and non-apoptotic (orange star) nuclei. The apoptotic cell is at the initiation stage, before force generation. (B) Box plot of Lamin::RFPt levels in apoptotic (n=11) and non-apoptotic (n=35) nuclei. Apoptotic nuclei were selected on their basal localization, during or just prior the force generation stage. Mann Whitney test p value < 0.0159 (*). (Related to Figure 2).

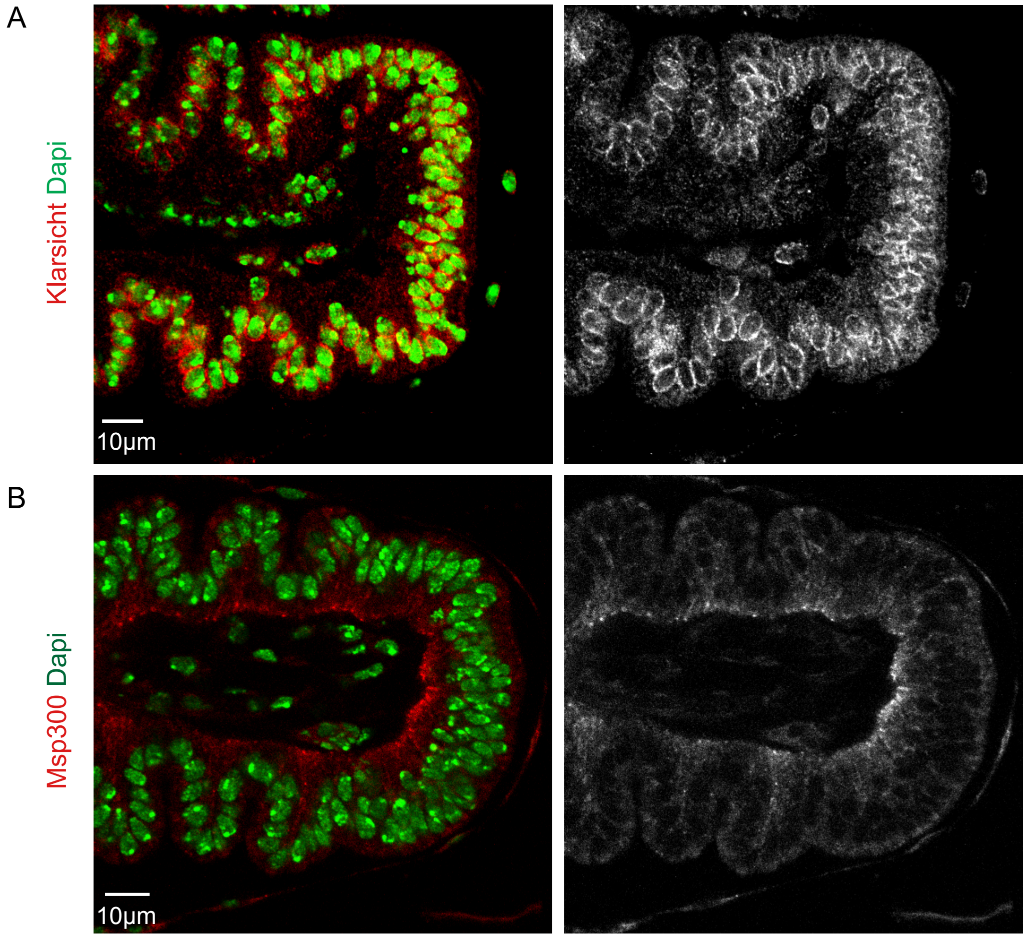

Figure S2. Localisation of KASH-domain proteins in the Drosophila leg
(A-B) Localization of the LINC members Klarsicht (A) and Msp-300 (B) in the leg tissue. Proteins of interest are shown in red and white and the nuclei in green. Msp-300 localisation is achieved using a Venus protein trap fly line. The Msp-300-Venus fusion protein is not perinuclear in the prepupal leg. By comparison, Klarsicht, which is detected by an antibody, is perinuclear in this tissue. Note that the black and white panel for Klarsicht in (A) is shown in purple in Fig. 3B. (Related to Fig. 3).

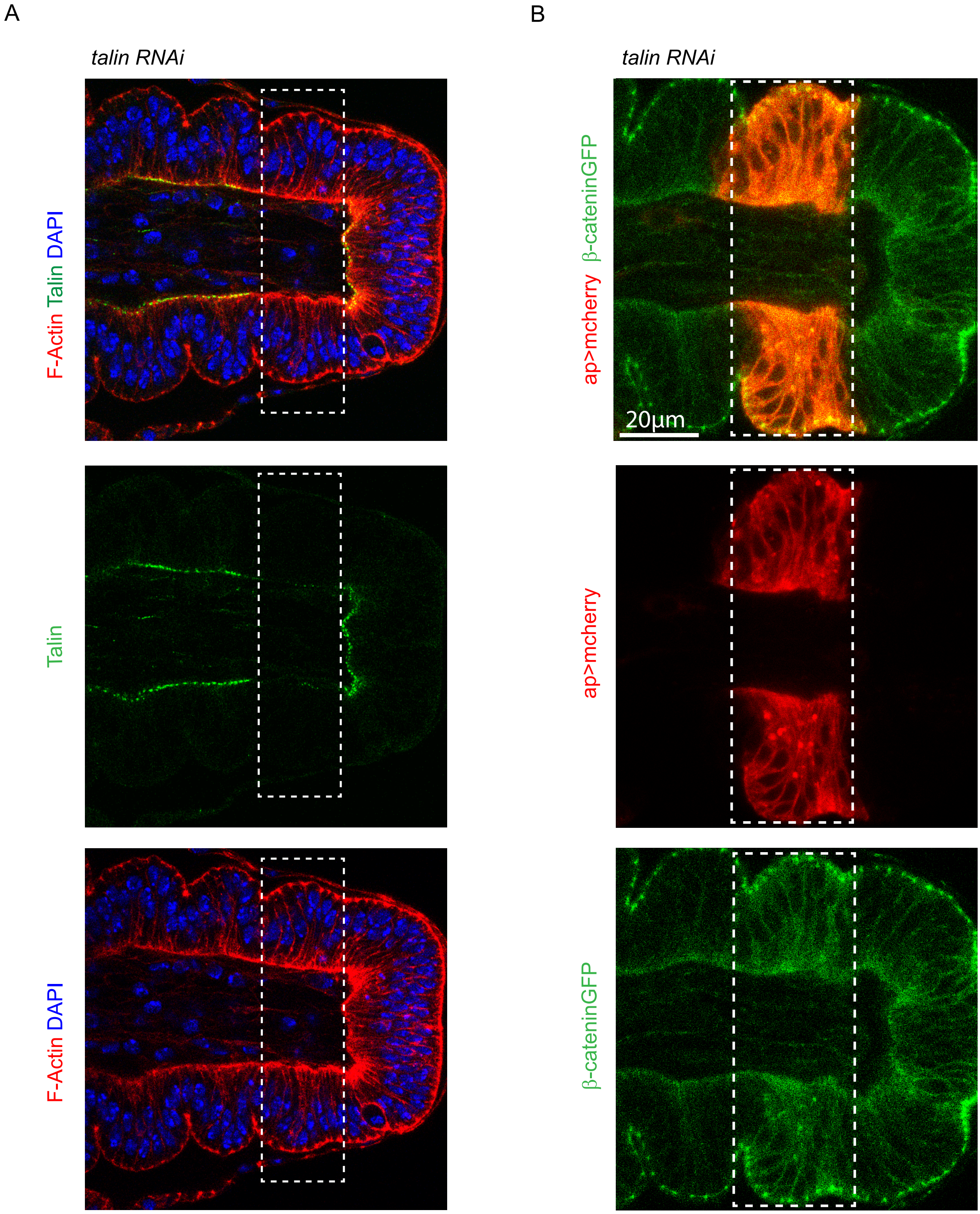

Figure S3. Talin inactivation does not alter tissue integrity in the prepupal leg
(A) Sagittal view of an apterous::Gal4; UAS::talin RNAi white pupa leg disc showing the distribution of Talin (green), F-actin organization (red) and nuclear positioning (blue). Note the strong reduction of Talin in the apterous domain (white dotted square). F-actin and nuclei positioning are unaltered in this domain. (B) Sagittal view of an apterous::Gal4; UAS::talin RNAi white pupal leg disc showing the distribution of β-catenin (green). Note that the epithelium is normally polarized in the apterous domain (white dotted square) in which *talin* is inactivated. UAS::mCherry labels the apterous domain. (Related to Figure 4)

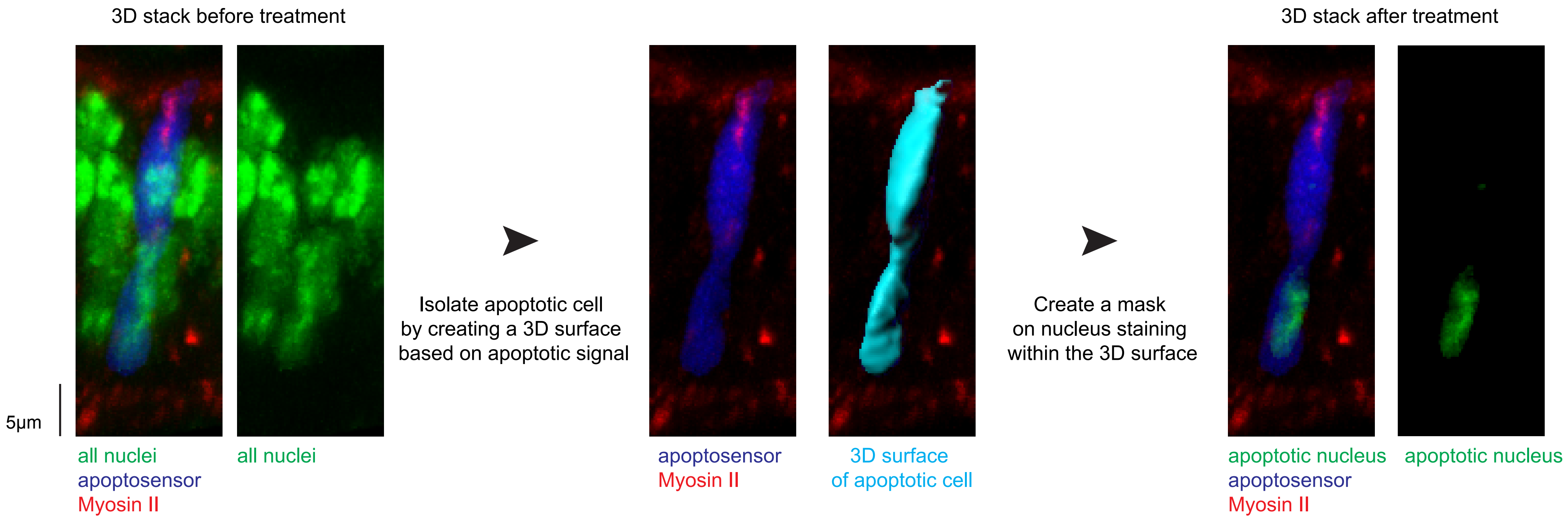

Figure S4. Post-acquisition method to isolate the apoptotic nucleus

Images recapitulating the different steps of apoptotic nucleus isolation from 3D reconstruction stack. 1. Isolation of an apoptotic cell by creating a 3D surface based on the expression of the apoptosensor (GC3Ai, blue). 2. Creation of a mask to extract nucleus staining (green) specifically in the apoptotic cell. Despite an originally crowded environment, this method allows to specifically visualize and track the apoptotic nucleus in live samples. (Related to Figure 5)
